# IRIS: Discovery of cancer immunotherapy targets arising from pre-mRNA alternative splicing

**DOI:** 10.1073/pnas.2221116120

**Published:** 2023-05-16

**Authors:** Yang Pan, John W. Phillips, Beatrice D. Zhang, Miyako Noguchi, Eric Kutschera, Jami McLaughlin, Pavlo A. Nesterenko, Zhiyuan Mao, Nathanael J. Bangayan, Robert Wang, Wendy Tran, Harry T. Yang, Yuanyuan Wang, Yang Xu, Matthew B. Obusan, Donghui Cheng, Alex H. Lee, Kathryn E. Kadash-Edmondson, Ameya Champhekar, Cristina Puig-Saus, Antoni Ribas, Robert M. Prins, Christopher S. Seet, Gay M. Crooks, Owen N. Witte, Yi Xing

**Affiliations:** ^a^Bioinformatics Interdepartmental Graduate Program, University of California, Los Angeles, CA 90095; ^b^Center for Computational and Genomic Medicine, The Children’s Hospital of Philadelphia, Philadelphia, PA 19104; ^c^Department of Microbiology, Immunology and Molecular Genetics, University of California, Los Angeles, CA 90095; ^d^Molecular Biology Institute, University of California, Los Angeles, CA 90095; ^e^Department of Molecular and Medical Pharmacology, David Geffen School of Medicine, University of California, Los Angeles, CA 90095; ^f^Graduate Group in Genomics and Computational Biology, University of Pennsylvania, Philadelphia, PA 19104; ^g^Eli and Edythe Broad Center of Regenerative Medicine and Stem Cell Research, University of California, Los Angeles, CA 90095; ^h^Department of Neurosurgery, David Geffen School of Medicine, University of California, Los Angeles, CA 90095; ^i^Division of Hematology-Oncology, Department of Medicine, David Geffen School of Medicine, University of California, Los Angeles, CA 90095; ^j^Jonsson Comprehensive Cancer Center, University of California, Los Angeles, CA 90095; ^k^Parker Institute for Cancer Immunotherapy, David Geffen School of Medicine, University of California, Los Angeles, CA 90095; ^l^Department of Surgery, David Geffen School of Medicine, University of California, Los Angeles, CA 90095; ^m^Department of Medicine, David Geffen School of Medicine, University of California, Los Angeles, CA 90095; ^n^Department of Pathology and Laboratory Medicine, David Geffen School of Medicine, University of California, Los Angeles, CA 90095; ^o^Division of Pediatric Hematology-Oncology, Department of Pediatrics, David Geffen School of Medicine, University of California, Los Angeles, CA 90095; ^p^Department of Pathology and Laboratory Medicine, Perelman School of Medicine, University of Pennsylvania, Philadelphia, PA 19104; ^q^Department of Biomedical and Health Informatics, The Children’s Hospital of Philadelphia, Philadelphia, PA 19104

**Keywords:** RNA splicing, immunotherapy, T cell receptors

## Abstract

Alternative splicing (AS) is prevalent in cancer, generating an extensive but largely unexplored repertoire of novel immunotherapy targets. We describe Isoform peptides from RNA splicing for Immunotherapy target Screening (IRIS), a computational platform capable of discovering AS-derived tumor antigens (TAs) for T cell receptor (TCR) and chimeric antigen receptor T cell (CAR-T) therapies. IRIS leverages large-scale tumor and normal transcriptome data and incorporates multiple screening approaches to discover AS-derived TAs with tumor-associated or tumor-specific expression. In a proof-of-concept analysis integrating transcriptomics and immunopeptidomics data, we showed that hundreds of IRIS-predicted TCR targets are presented by human leukocyte antigen (HLA) molecules. We applied IRIS to RNA-seq data of neuroendocrine prostate cancer (NEPC). From 2,939 NEPC-associated AS events, IRIS predicted 1,651 epitopes from 808 events as potential TCR targets for two common HLA types (A*02:01 and A*03:01). A more stringent screening test prioritized 48 epitopes from 20 events with “neoantigen-like” NEPC-specific expression. Predicted epitopes are often encoded by microexons of ≤30 nucleotides. To validate the immunogenicity and T cell recognition of IRIS-predicted TCR epitopes, we performed in vitro T cell priming in combination with single-cell TCR sequencing. Seven TCRs transduced into human peripheral blood mononuclear cells (PBMCs) showed high activity against individual IRIS-predicted epitopes, providing strong evidence of isolated TCRs reactive to AS-derived peptides. One selected TCR showed efficient cytotoxicity against target cells expressing the target peptide. Our study illustrates the contribution of AS to the TA repertoire of cancer cells and demonstrates the utility of IRIS for discovering AS-derived TAs and expanding cancer immunotherapies.

## Significance

Despite the success of cancer immunotherapy, discovering actionable tumor antigens as immunotherapy targets remains a major challenge. Aberrant alternative splicing (AS) is widespread in cancer and generates a large repertoire of potential immunotherapy targets. However, there is no well-established strategy to discover AS-derived immunotherapy targets. We describe an integrated computational workflow for comprehensive discovery and characterization of AS-derived immunotherapy targets, leveraging large-scale RNA-seq resources of tumor and normal tissues. We demonstrate the application of this workflow for target discovery of neuroendocrine prostate cancer, a highly lethal cancer with no effective therapies. We experimentally confirm the immunogenicity and T cell recognition of AS-derived T cell receptor targets. Collectively, this work introduces a broadly applicable framework for discovering cancer immunotherapy targets arising from AS.

Cancer immunotherapy has gained remarkable success in the past decade. Checkpoint inhibitors, like neutralizing PD-1 and CTLA-4 antibodies, are thought to be clinically effective by reactivating tumor-specific T cells (1). In contrast, adoptive cell therapies use genetically modified T cell receptors (TCRs) and chimeric antigen receptor T cells (CAR-T) to target antigens expressed in cancer cells (2). The insight that cancer cells express specific T cell-reactive antigens has galvanized antigen discovery efforts in recent years (3–6). Nevertheless, the discovery of tumor antigens (TAs) remains a major challenge (7, 8). Although somatic mutation-derived TAs have been successfully targeted by cancer therapies (9–12), this approach remains largely ineffective for tumors with low or moderate mutation load (3).

Post-transcriptional RNA processing is an essential layer of eukaryotic gene expression, and its dysregulation has a major impact on the cancer cell proteome (13–15). Various types of RNA-level dysregulation can generate aberrant proteins and immunogenic peptides in cancer cells (16–20). In a pancancer analysis, Kahles *et al*. found that tumors harbor up to 30% more alternative splicing (AS) events than normal tissues, and some of the resulting peptides are predicted to be presented by HLA molecules (16). In another study, experimental evidence of HLA class I (HLA-I) presentation of peptides derived from intron retention, a specific type of AS, was reported based on mass spectrometry (MS) proteomics data (17). These findings have inspired a growing interest in AS as a rich source of potential immunotherapy targets (14, 21).

Currently, there are limited computational tools for discovering AS-derived TAs. Two recently published tools, ASNEO (22) and NeoSplice (23), sought to discover AS-derived TCR targets for cancer immunotherapy. Both tools use RNA-seq data of tumor tissues as well as selected normal tissues to identify putative tumor-specific AS events, followed by HLA binding prediction. However, they lack the computational infrastructure to leverage large cohorts of tumor and normal transcriptomes in public repositories to comprehensively determine the tumor association and specificity of predicted targets. Importantly, neither study experimentally tested the immunogenicity of predicted targets or their ability to activate functional T cell responses.

**Fig. 1.**
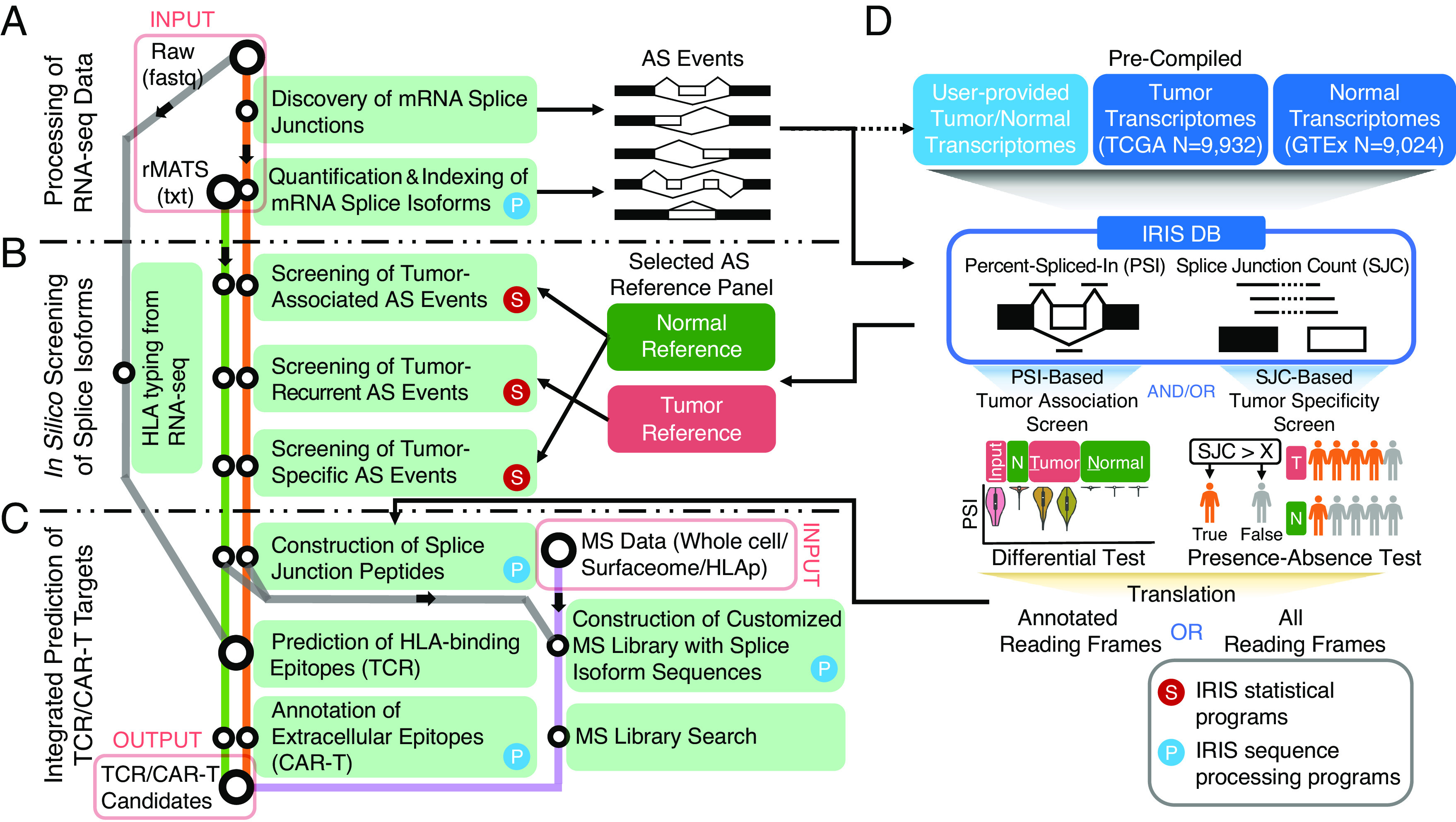
IRIS: A big-data informed computational platform for discovering AS-derived cancer immunotherapy targets. Overall workflow of IRIS, computational modules, large-scale reference database of AS profiles, and screening tests are illustrated. IRIS has three main computational modules: (*A*) RNA-seq data processing, (*B*) in silico screening, and (*C*) TCR/CAR-T target prediction. A flowchart illustrates the key components and analytical steps of IRIS. (*D*) Illustration of IRIS DB, a reference database of AS profiles across tumor and normal tissue samples, and two screening tests to assess tumor association and specificity. AS, alternative splicing; TCR, T cell receptor; CAR-T, chimeric antigen receptor T cell.

We have developed an in silico platform to discover and prioritize AS-derived immunotherapy targets of varying degrees of tumor association and specificity, by utilizing an “AS reference” that represents splicing profiles of tens of thousands of tumor and normal transcriptomes generated by large-scale consortia (e.g. GTEx, TCGA) (24, 25). Our platform, Isoform peptides from RNA splicing for Immunotherapy target Screening (IRIS), enables a big-data informed discovery of AS-derived TCR and CAR-T targets through a streamlined framework (Fig. 1). IRIS is powered by the new generation of our widely used rMATS software (rMATS-turbo) (26) for AS analysis of RNA-seq data, with a substantial improvement in speed and computational efficiency enabling ultrafast analyses of AS events across massive RNA-seq datasets. We initially tested the utility of IRIS through a proof-of-concept analysis using immunopeptidomics data of human cell lines. We then applied IRIS to RNA-seq data of neuroendocrine prostate cancer (NEPC), a metastatic and highly lethal prostate cancer with no effective long-term treatments or targeted therapies (27). To validate the immunogenicity and T cell recognition of IRIS-predicted TCR targets, we performed in vitro T cell priming in combination with single-cell TCR sequencing, followed by reconstitution and functional characterization of TCRs transduced into human peripheral blood mononuclear cells (PBMCs). Collectively, our study illustrates the contribution of AS to the TA repertoire of cancer cells and demonstrates the utility of IRIS for discovering AS-derived TAs and expanding cancer immunotherapies.

## Results

### Overall Design of the IRIS Computational Framework.

To identify AS-derived immunotherapy targets, IRIS incorporates three main modules: processing of RNA-seq data, in silico screening for tumor-associated or tumor-specific AS events, and integrated prediction and prioritization of TCR and CAR-T targets (Fig. 1). Briefly, IRIS first discovers and quantifies various types of AS events from user-provided RNA-seq data of a given tumor type (Fig. 1*A*). Then, AS events are fed into an in silico screening module to identify tumor-associated or tumor-specific events, based on a comparison against large-scale reference RNA-seq resources of tumor and normal tissues (Fig. 1*B*). Lastly, IRIS performs TCR and CAR-T target prediction for the identified AS events (Fig. 1*C*).

In IRIS’s RNA-seq data processing module, user-provided RNA-seq data of a given tumor type are analyzed by the rMATS-turbo software to comprehensively discover and quantify AS events corresponding to major types of AS patterns (Fig. 1*A*). The rMATS/rMATS-turbo software was developed by our group for AS analysis of RNA-seq data and has been widely used by the research community since 2014 (26, 28). Compared to the original rMATS software (28), rMATS-turbo incorporates a refactored computational workflow with substantially improved data processing speed and efficiency, allowing it to scale up to massive RNA-seq datasets with tens of thousands of samples (26). Powered by the speed and efficiency of rMATS-turbo, we uniformly processed 18,956 RNA-seq samples in public data repositories generated by large-scale consortia (TCGA, GTEx), representing 33 tumor types and 51 normal tissue types of 30 histological sites (*SI Appendix*, Fig. S1 and Table S1). Results of this analysis were organized into the IRIS Alternative Splicing Database (IRIS DB), which contains ratio-based [percent-spliced-in (PSI)] (29) and count-based [splice junction (SJ) read count] quantification of all major types of AS events detected in TCGA and GTEx (Fig. 1*D*). The IRIS DB is indexed, allowing for efficient query of AS events in large-scale tumor and normal transcriptomes from diverse tumor types and tissue origins.

IRIS’s in silico screening module provides three distinct screening tests to identify targets of varying degrees of tumor association and specificity (Fig. 1*B*). Specifically, IRIS compares AS events from user-provided RNA-seq data of tumor samples to a reference panel of user-specified tumor and normal tissues selected from the IRIS DB. The default “tumor-association screen” uses the PSI metric to identify tumor-associated AS events, via a differential AS (PSI value) analysis between tumor and normal tissues based on various user-defined criteria, such as p-value and change of PSI value (delta PSI), as well as fold-change (FC) of tumor-enriched isoform (*Materials and Methods*). Moreover, to identify AS events with “neoantigen-like” tumor specificity, defined as AS-derived SJs that are exclusively expressed in tumor tissues, IRIS performs a more stringent “tumor-specificity screen” by testing and comparing the presence-absence of a given SJ between tumor and normal tissues. Specifically, for each sample group (e.g., user-provided RNA-seq data of tumor samples, or a reference normal tissue type in the IRIS DB), IRIS calculates the percentage of samples expressing a given SJ of interest at or above a user-defined read count threshold. IRIS then performs a Fisher Exact test to identify “tumor-specific” SJs that are expressed in a significantly higher percentage of tumor samples than in normal tissue samples. IRIS reports a tumor-associated AS event as tumor-specific if all SJ(s) of its corresponding tumor-enriched isoform are tumor-specific as defined by the tumor-specificity screen. Finally, IRIS also incorporates a “tumor-recurrence screen” to compare AS events between user-provided RNA-seq samples of a given tumor type to user-selected tumor types of similar histology in the IRIS DB. This test allows IRIS to identify AS events that are recurrent (shared) among independent cohorts of the similar tumor type.

IRIS’s target prediction module incorporates various prediction tools and annotation resources to identify candidate targets for immunotherapies (Fig. 1*C*). The module first constructs SJ peptides of identified AS events, and then predicts AS-derived targets for TCR or CAR-T therapies (*Materials and Methods*). The TCR target prediction function first performs tumor HLA typing using RNA-seq data or accepts user-specified HLA types, then integrates multiple HLA binding prediction algorithms for predicting TCR targets and/or peptide vaccines. Specifically, IRIS uses Immune Epitope Database (IEDB) (30) predictors to obtain the putative HLA binding affinities of candidate peptides. The IEDB “recommended” mode runs multiple prediction tools to generate multiple predictions of binding affinity, which IRIS summarizes as a median IC_50_ value. In parallel, the CAR-T target prediction function maps AS-derived peptides to protein extracellular domain annotations curated by UniProtKB (31) (*SI Appendix*, Fig. S2). IRIS also includes an option to confirm predicted AS-derived targets using MS data via proteotranscriptomics data integration (Fig. 1*C*). This option provides an orthogonal approach for target discovery and validation by integrating RNA-seq data with various types of MS data, such as whole-cell proteomics, surfaceomics, or immunopeptidomics data. Specifically, IRIS builds a custom library of AS-derived peptides and then searches MS spectra against this library, allowing proteomic validation of AS-derived targets using MS data.

### AS-Derived Peptides Are Present in Cell Line Immunopeptidomes.

**Fig. 2.**
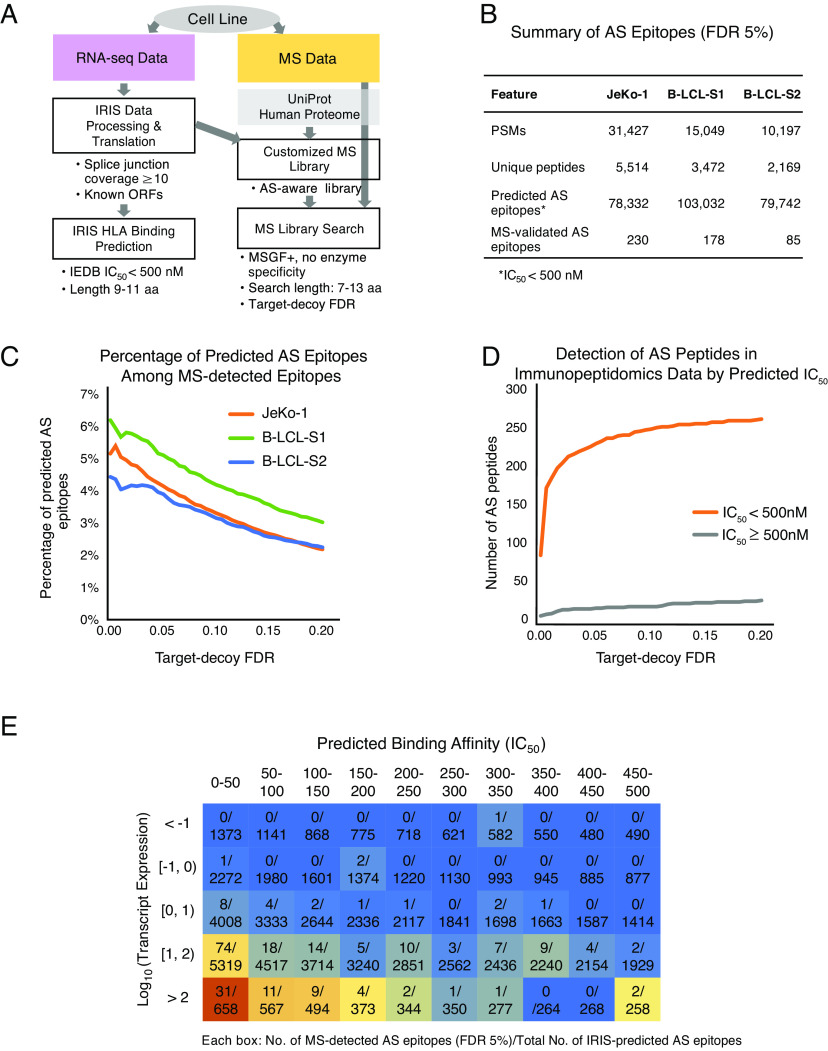
Proteotranscriptomics analysis of AS-derived peptides in cell line immunopeptidomes. (*A*) Proteotranscriptomics workflow adopted by IRIS for discovering AS-derived peptides in MS-based proteomics datasets. IRIS accepts various types of MS data (*Right*), such as whole-cell proteomics, surfaceomics, or immunopeptidomics (HLA peptidomics) data. Aided by RNA-seq data (*Left*), an RNA-seq-augmented custom proteome library is constructed and searched using MSGF+. (*B*) Summary of AS-derived epitopes in JeKo-1 and B-LCL cell lines. Peptide-spectrum matches (“PSMs”) and “Unique peptides” are provided by MSGF+ with a target-decoy FDR of 5%. “Predicted AS epitopes” are generated by the IRIS target prediction module, which utilizes IEDB predictors. “MS-validated AS epitopes” are defined as epitopes that are predicted by IRIS and detected in the immunopeptidomics data. (*C*) Percentage of IRIS-predicted AS-derived epitopes among all MS-detected epitopes for three cell lines. Graph shows the percentage of all MS-detected epitopes that are IRIS-predicted AS-derived epitopes (*y*-axis) as a function of the MSGF+ target-decoy FDR (*x*-axis). (*D*) Preferential detection of high-affinity AS-derived peptides in immunopeptidomics data. Graph shows the number of AS-derived peptides detected in JeKo-1 immunopeptidomics data (*y*-axis) as a function of the MSGF+ target-decoy FDR (*x*-axis). Peptides with high (IC_50_ < 500 nM; orange) and low (IC_50_ ≥ 500 nM; gray) predicted HLA binding affinities are shown. (*E*) Heatmap illustration of the distribution of AS-derived peptides detected in JeKo-1 immunopeptidomics data as a function of predicted HLA binding affinity and transcript expression level. AS-derived peptides are binned by their corresponding transcript expression levels and IEDB-predicted HLA binding affinities. Heatmap is colored from red (high) to blue (low), reflecting the proportion of IRIS-predicted AS-derived epitopes that are MS-detected in each bin.

In a proof-of-concept analysis, we sought to identify AS-derived peptides that are presented by HLA molecules, i.e., AS-derived epitopes, by applying IRIS’s RNA-seq data processing and target prediction modules to RNA-seq and MS-based immunopeptidomics data of multiple cell lines (Fig. 2*A*). Specifically, we analyzed paired RNA-seq and immunopeptidomics data of two B lymphoblastoid cell lines (B-LCL) (32) and one cancer cell line (JeKo-1 lymphoma) (33). Focusing on predicting HLA-I binding to AS-derived peptides, we found 230, 178, and 85 peptides present in the immunopeptidomics data of JeKo-1, B-LCL-S1, and B-LCL-S2, respectively, after controlling for the target-decoy false discovery rate (FDR) at 5% (Fig. 2*B* and Dataset S1). Our results provide evidence that AS-derived peptides are presented by HLA-I molecules.

We assessed the concordance between AS-derived epitopes predicted by HLA binding algorithms (“IEDB recommended”, see *Materials and Methods*) and those detected from immunopeptidomics data. For all three cell lines, the percentage of AS-derived epitopes among all epitopes detected from immunopeptidomics data increased progressively with more stringent target-decoy FDR cutoffs (Fig. 2*C*). Among all epitopes detected from immunopeptidomics data, AS-derived peptides with high predicted HLA binding affinities (IC_50_ < 500 nM) substantially outnumbered AS-derived peptides with low predicted HLA binding affinities (IC_50_ ≥ 500 nM) (see Fig. 2*D* for data on JeKo-1). Moreover, in all three cell lines, we observed an increase in the fraction of AS-derived epitopes detected from immunopeptidomics data as a function of higher transcript expression levels and higher predicted HLA binding affinities (see Fig. 2*E* for data on JeKo-1). For example, only 52 out of 56,254 IRIS-predicted AS-derived epitopes were detected from immunopeptidomics data when the transcript expression level was lower than 10 fragments per kilobase million (FPKM) or the predicted HLA binding affinity was weaker than IC_50_ of 250 nM. In contrast, 178 out of 22,077 AS-derived epitopes were detected from immunopeptidomics data when the transcript expression level was higher than 10 FPKM and the predicted HLA binding affinity was stronger than IC_50_ of 250 nM. The largest fraction of AS-derived epitopes detected from immunopeptidomics data was observed in the bottom leftmost bin of Fig. 2*E*. This bin represents AS-derived epitopes with the highest transcript expression level (>100 FPKM) and strongest predicted HLA binding affinity (IC_50_ < 50 nM). Our results demonstrate that AS-derived epitopes supported by immunopeptidomics data are enriched for transcripts with high expression levels and peptides with strong predicted HLA binding affinities, consistent with the expected pattern of HLA-epitope binding (34).

### Discovery of AS-Derived Immunotherapy Targets for NEPC.

**Fig. 3.**
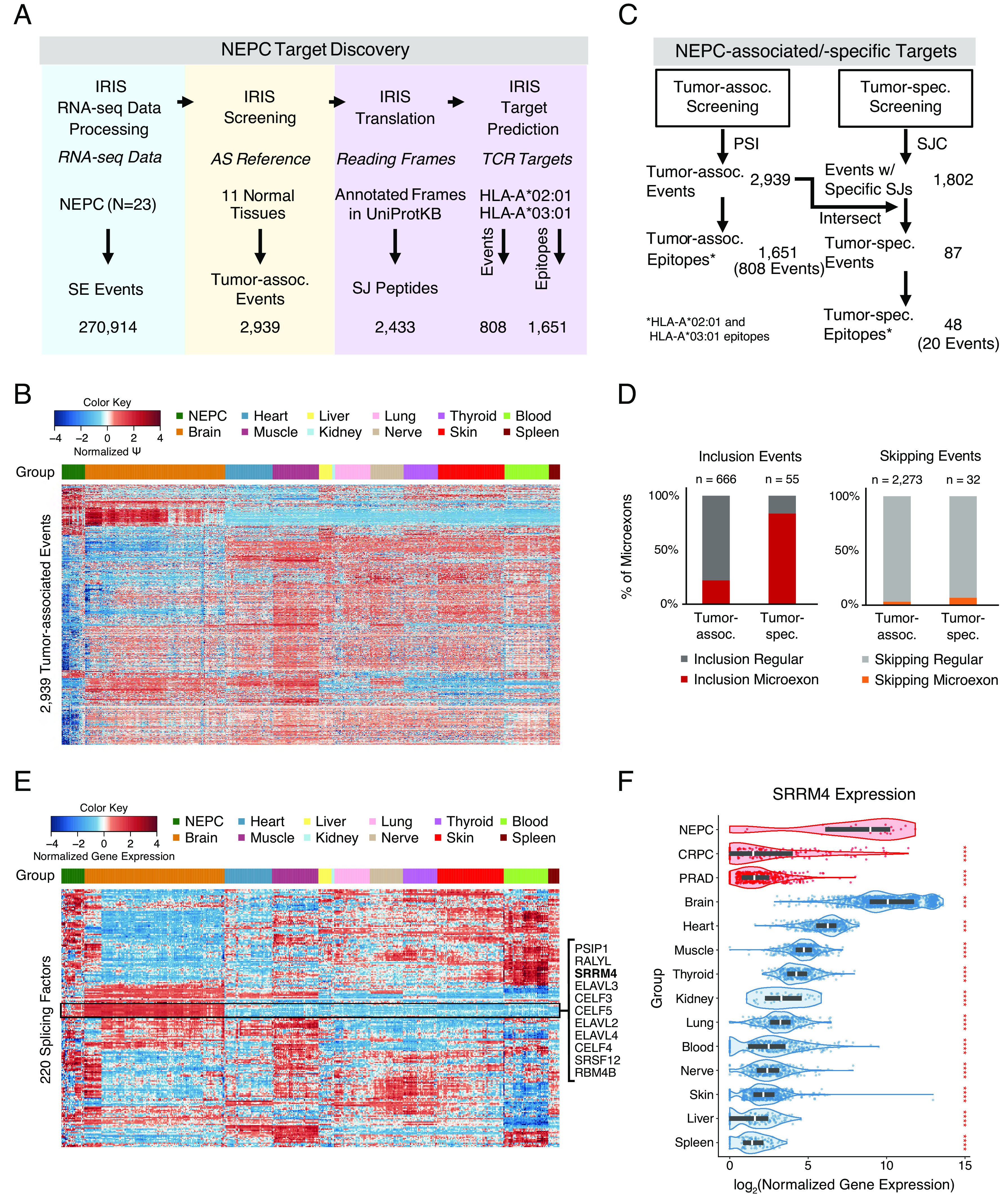
IRIS discovery of AS-derived targets for NEPC. (*A*) Stepwise results of IRIS to identify AS-derived cancer immunotherapy targets from 23 NEPC samples. Skipped exon (SE) events identified by the IRIS RNA-seq data processing module were screened against 11 normal tissue types from the IRIS DB to identify tumor-associated events and predict corresponding TCR targets. (*B*) Heatmap of AS profiles of 2,939 NEPC-associated SE events across NEPC and 11 normal tissue types. (*C*) Summary of NEPC-associated/-specific targets. “Events w/ Specific SJs” are SE events that contain tumor-specific SJ(s) identified from the SJ count (SJC)-based tumor-specificity screen. (*D*) Bar plots showing the percentage of NEPC-associated and NEPC-specific SE events involving inclusion (*Left*) or skipping (*Right*) of microexons in NEPC. (*E*) Heatmap of gene expression levels of 220 splicing factors across NEPC and 11 normal tissue types. (*F*) Violin plots of log-transformed gene expression levels of serine/arginine repetitive matrix protein 4 (SRRM4) across NEPC, metastatic castration-resistant prostate cancer (CRPC), primary prostate adenocarcinoma (PRAD), and 11 normal tissue types. Two-sided Mann–Whitney *U* test was conducted to compare SRRM4 gene expression levels between NEPC and every other group. Red asterisks represent *P*-values (*: *P* ≤ 0.05, **: *P* ≤ 0.01, ***: *P* ≤ 0.001, ****: *P* ≤ 0.0001). Groups are colored by tumor (red) and normal (blue) tissue.

To demonstrate the utility of IRIS in discovering AS-derived immunotherapy targets in tumor specimens, we applied IRIS to a published RNA-seq dataset of 23 NEPC samples (Fig. 3*A*). For the normal tissue panel, we selected 11 vital tissues from the IRIS DB. In total, 270,914 skipped exon (SE) events were identified and quantified in the NEPC dataset (Fig. 3*A*; blue panel). Using the PSI-based tumor-association screen, IRIS identified 2,939 SE events as tumor-associated (Fig. 3*A*; yellow panel and Dataset S2a). As illustrated in Fig. 3*B*, hierarchical clustering based on PSI values confirmed that NEPC-associated SE events have distinct splicing profiles in most of the normal tissue types and modestly similar splicing profiles in the normal brain as compared to the splicing profiles in NEPC.

Next, for each NEPC-associated SE event, SJ(s) of the tumor-enriched isoform were translated into peptides, followed by TCR target prediction (Fig. 3*A*; purple panel). From 2,939 NEPC-associated SE events, 2,433 tumor-enriched SJs can be translated into peptide sequences based on annotated reading frames. Of these, 1,651 epitopes from 808 NEPC-associated SE events were predicted as TCR targets for two common HLA types, HLA-A*02:01 and HLA-A*03:01 (Dataset S2b). Using the same procedure, we predicted 385 epitopes from 207 NEPC-associated AS events corresponding to alternative 5′ splice sites (A5SS), alternative 3′ splice sites (A3SS), and retained introns (RI) as additional TCR targets (*SI Appendix*, Figs. S3 and Dataset S2 d, e, g, h, j, and k).

IRIS also identifies tumor-associated SJ peptides located in annotated extracellular regions of cell-surface proteins. In total, 168 NEPC-associated AS events, including 119 SE events (five examples are shown in *SI Appendix*, Fig. S4), were identified as located in annotated extracellular regions of cell-surface proteins (*SI Appendix*, Fig. S3 and Dataset S2 c, f, i, and l). Such events may represent potential CAR-T targets.

### NEPC-Specific SE Events Are Enriched for Microexons.

To prioritize targets with greater tumor specificity, we used IRIS to perform a more stringent tumor-specificity screen by testing and comparing the presence-absence of a given SJ between tumor and normal tissues (Fig. 3*C*). This screen identified 1,802 SE events with NEPC-specific SJs. Intersecting these events with the 2,939 NEPC-associated SE events identified by the tumor-association screen yielded a prioritized set of 87 NEPC-specific SE events that could potentially produce “neoantigen-like” AS-derived targets. Of the NEPC-specific peptides encoded by these events, 48 epitopes from 20 events were predicted to bind to HLA-A*02:01 or HLA-A*03:01.

Additionally, we performed an optional secondary tumor-association screen, based on normalized SJ read counts in the unit of counts per million (CPM) (*Materials and Methods*). This screening test directly compares the expression level of a given SJ between tumor and normal tissues, thus accounting for the joint effects of overall gene expression and AS. We found that 1,317 of the 2,939 NEPC-associated events identified by the default PSI value-based tumor-association screen also passed this secondary tumor-association screen based on SJ CPM values. Moreover, 78 of the 87 NEPC-specific events identified by the tumor-specificity screen also passed this secondary tumor-association screen.

We found that these 87 NEPC-specific SE events were significantly enriched for events corresponding to NEPC-specific inclusion of microexons [i.e., exons no more than 30 nucleotides in length (35)] (Fig. 3*D*). Among the 55 events corresponding to NEPC-specific exon inclusion, 46 (83.6%) involved a microexon. Among the 666 events corresponding to NEPC-associated exon inclusion, 145 (21.8%) involved a microexon (Fig. 3*D*; *Left*). In contrast, the percentage of events involving microexons was much lower for those corresponding to NEPC-specific or NEPC-associated exon skipping [2 out of 32 (6.3%) and 56 out of 2,273 (2.5%), respectively; Fig. 3*D*; *Right*]. To investigate whether NEPC-specific microexon inclusion is correlated with the expression of splicing factors, we examined gene expression levels of 220 splicing factors (36) across NEPC and the normal tissue panel. Hierarchical clustering of splicing factor gene expression levels revealed a cluster of 11 splicing factors with elevated expression in NEPC and the normal brain compared to the rest of the normal tissue panel (Fig. 3*E*). Notably, serine/arginine repetitive matrix 4 (SRRM4), which was previously reported to promote neuronal-specific inclusion of microexons through an evolutionarily conserved mechanism (35), was among the 11 splicing factors overexpressed in NEPC and the normal brain. Further comparison of SRRM4 gene expression levels among NEPC, metastatic castration-resistant prostate cancer (CRPC), and primary prostate adenocarcinoma (PRAD) samples revealed that overexpression of SRRM4 is unique to NEPC (Fig. 3*F*), a finding that is consistent with a previous report (37). Together, these observations point to SRRM4, among other splicing factors, as a likely contributor to NEPC-specific inclusion of microexons and consequently the TA repertoire of NEPC.

### Big-Data Informed Evaluation and Visualization of AS-Derived Immunotherapy Targets.

**Fig. 4.**
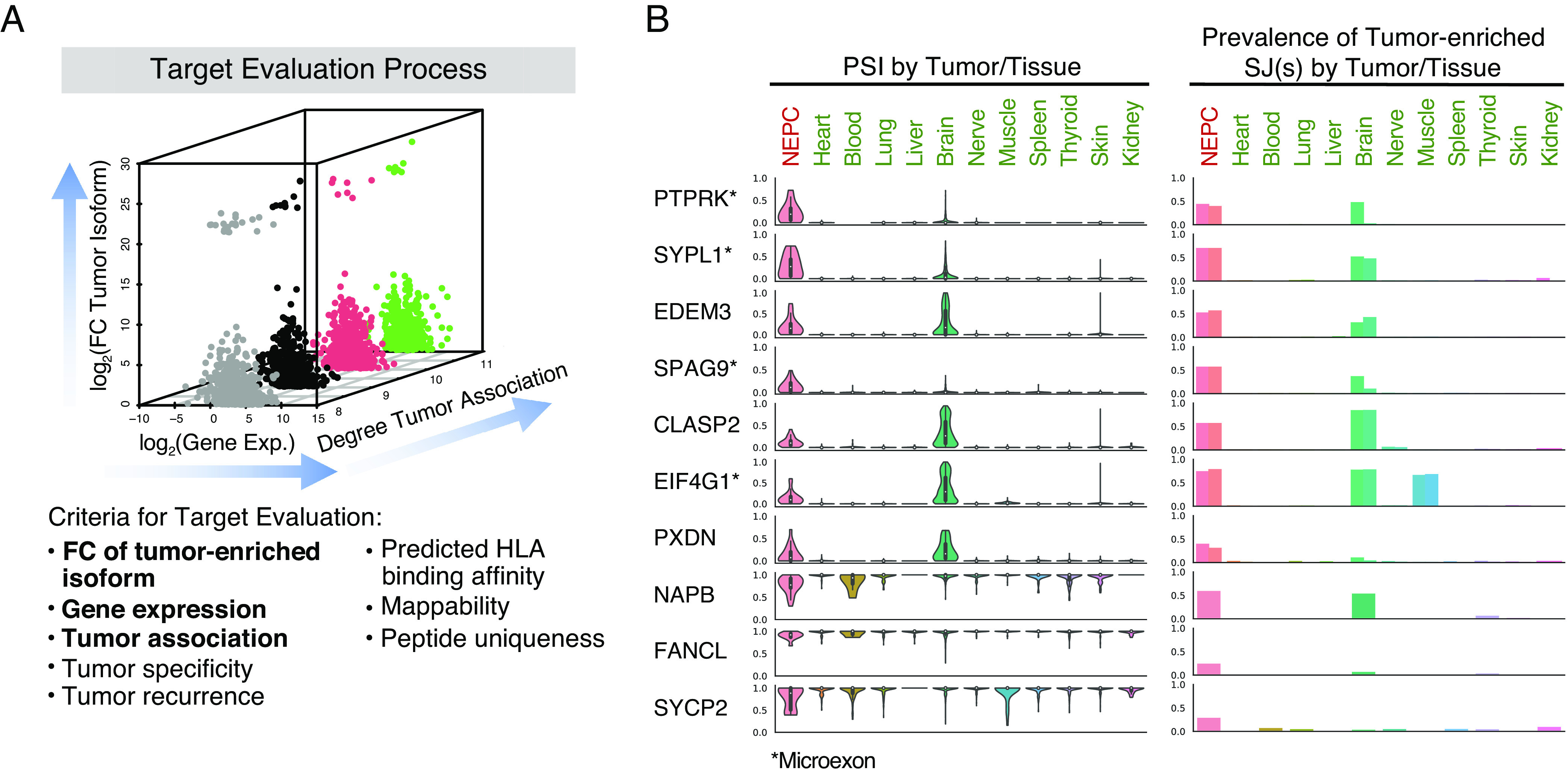
Evaluation and visualization of IRIS-predicted targets for NEPC. (*A*) The target evaluation process for NEPC. The three-dimensional scatterplot illustrates the three main criteria used to evaluate IRIS-predicted targets: degree of tumor association, FC of the tumor-enriched isoform between tumor and normal tissues, and gene expression level in tumor tissues. These and additional criteria to evaluate targets are listed below the scatterplot. Criteria illustrated in the scatterplot are bolded. (*B*) Representative examples of 10 IRIS-predicted TCR targets are visualized by IRIS in paired violin and bar plots. Each row shows one IRIS-predicted TCR target. Violin plots show the PSI values of each target in NEPC and the normal tissue panel. Bar plots show the fraction of samples expressing the SJ(s) of the tumor-enriched isoform in NEPC and the normal tissue panel. If the tumor-enriched isoform is the exon inclusion isoform, the bar plot displays the upstream and downstream inclusion SJ as two bars. If the tumor-enriched isoform is the exon skipping isoform, the bar plot displays the skipping SJ as one bar.

IRIS generates an integrated report that allows researchers to evaluate and visualize predicted targets based on multiple criteria (Fig. 4). The three main criteria are: degree of tumor association, FC of the tumor-enriched isoform between tumor and normal tissues, and gene expression level in tumor tissues (see Fig. 4*A* for visualization of these criteria for predicted tumor-associated NEPC targets). The “degree of tumor association” is defined as the number of normal tissue types compared to which the tumor tissues have consistently and significantly higher or lower PSI values. The “FC of tumor-enriched isoform” is calculated as the fold-change of the proportion of the tumor-enriched isoform in tumor tissues over the average proportion of the tumor-enriched isoform in all normal tissue types of the normal tissue panel. The gene expression level is the median gene expression level of the corresponding gene in tumor tissues. IRIS also reports additional features for predicted targets, including tumor specificity, predicted HLA binding affinity, as well as various genome or protein annotations [e.g., mappability, peptide uniqueness, etc. (*SI Appendix*, *Supplementary Materials and Methods*)].

Representative examples of 10 NEPC-associated TCR targets are shown in paired violin and bar plots generated by IRIS (Fig. 4*B*; the first 8 are also NEPC-specific). To illustrate tumor association, violin plots show the PSI value of each target in NEPC and the normal tissue panel (Fig. 4*B*; *Left*). To visualize tumor specificity, bar plots show the fraction of samples expressing the SJ(s) of the tumor-enriched isoform in NEPC and the normal tissue panel (Fig. 4*B*; *Right*). As expected, predicted TCR targets display distinct splicing profiles in NEPC relative to most of the normal tissue types, with the occasional exception being the normal brain. For example, an SE event in protein tyrosine phosphatase receptor type K (PTPRK) is selected by both tumor-association and tumor-specificity screens, with the tumor-enriched isoform including a microexon. Its tumor association is reflected by violin plots of PSI values, showing that the SE event has an average PSI value of 27% among NEPC samples as compared to almost 0% (no exon inclusion) across the normal tissue panel. The bar plots show that the two SJs of the exon included isoform are present in approximately half of NEPC samples, whereas they are absent in nearly all tissue types in the normal tissue panel except for one SJ in the normal brain. Likewise, a known microexon target of SRRM4 in eukaryotic translation initiation factor 4 gamma 1 (EIF4G1) (38) exhibits elevated exon inclusion in NEPC (and in the normal brain), as shown by both screens (Fig. 4*B*). To facilitate data exploration and visualization, we developed IRIS Explorer (https://xingshiny2.research.chop.edu/shiny/IRIS/), a web-based tool to explore and visualize IRIS results (*SI Appendix*, Fig. S5).

### Isolation and Characterization of TCRs Reactive to IRIS-Predicted NEPC Epitopes.

From 1,651 NEPC-associated epitopes, 76 unique epitopes were selected from 216 epitopes that met additional criteria for FC of the tumor-enriched isoform and gene expression level in tumor tissues and had predicted HLA-A*02:01 binding affinity < 500 nM (30). These epitopes were selected as candidates to study their immunogenicity and identify their cognate TCRs (*Materials and Methods* and Dataset S3a).

**Fig. 5.**
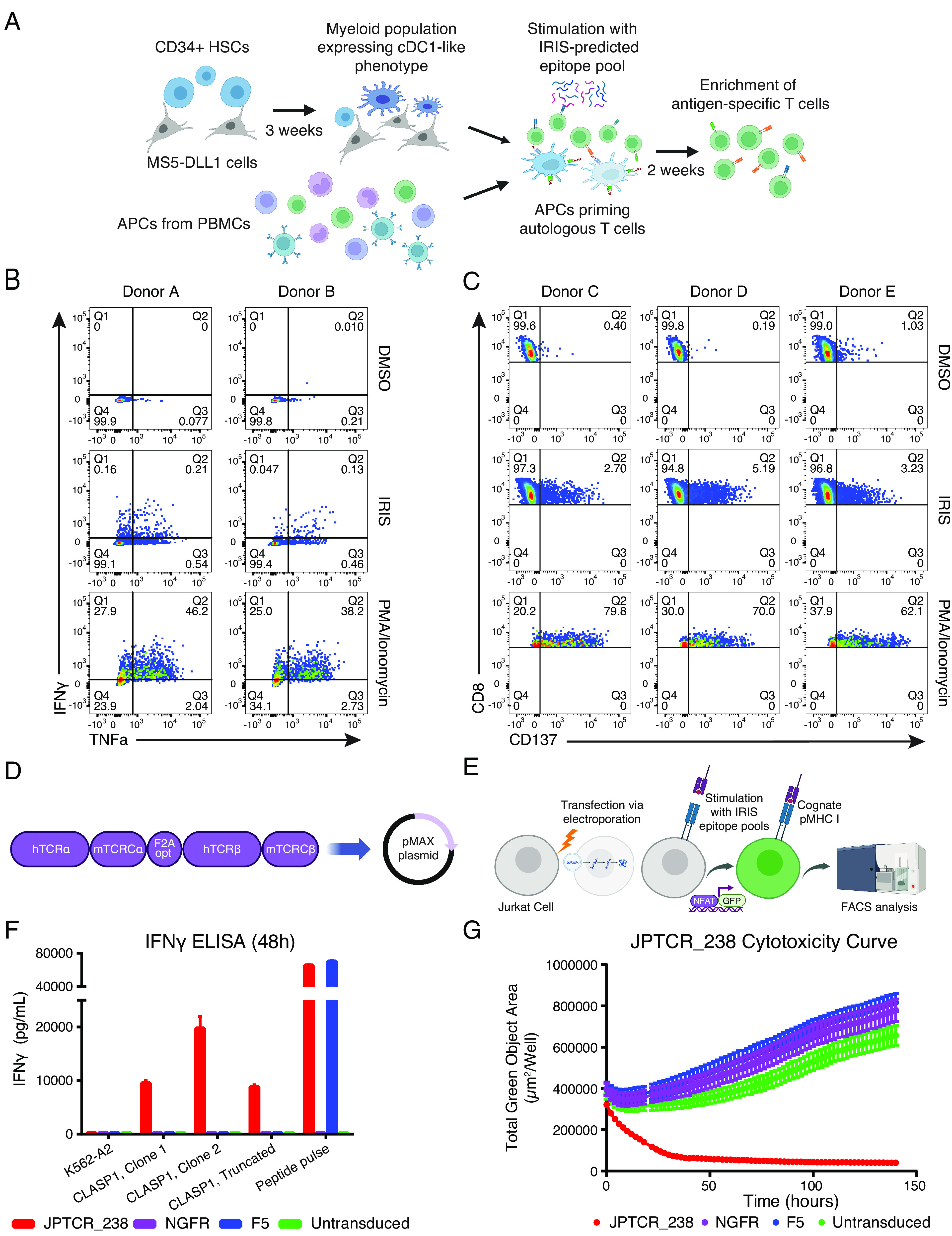
Isolation and characterization of TCRs reactive to IRIS-predicted NEPC epitopes. (*A*) IRIS-epitope priming using two APC systems: (1) conventional type 1 dendritic cell (cDC1)-like cells differentiated from autologous CD34+ hematopoietic stem cells (HSCs), and (2) existing APCs from PBMCs. (*B*) Example of reactive T cell populations primed with a DMSO negative control, IRIS epitope pool, or PMA/Ionomycin using the CLInt-seq TNFα/IFNγ intracellular marker staining strategy. (*C*) Example of reactive T cell populations primed with a DMSO negative control, IRIS epitope pool, or PMA/Ionomycin by the CD137 surface marker staining strategy. (*D*) Overview of the cloning strategy for TCRα/β chains in the pMAX system for Jurkat-NFAT-GFP screening. (*E*) Overview of the Jurkat-NFAT-GFP reporter system. (*F*) IFNγ ELISA of one specific TCR (JPTCR_238) targeting an IRIS-predicted AS-derived epitope in CLASP1, when co-cultured with K562-A2-GFP single-cell clones transduced to express a full-length or truncated CLASP1 protein isoform. Error bars indicate SD (n = 3). JPTCR_238: an isolated TCR targeting an IRIS-predicted epitope in CLASP1; F5: a clinically tested TCR targeting the MART1 melanoma antigen; NGFR: empty vector with no introduced TCR as a negative control; Untransduced: untransduced as a negative control. (*G*) Cytotoxicity analysis by live cell imaging of a K562-A2-GFP single-cell clone transduced with a full-length CLASP1 protein isoform containing an IRIS-predicted epitope targeted by JPTCR_238. F5 TCR, NGFR (no TCR introduced), and untransduced were used as negative controls.

To expand and isolate cognate T cells targeting predicted epitopes, PBMCs were stimulated with exogenously added peptides using two types of antigen-presenting cell (APC) systems, including: 1) dendritic cells (DCs) differentiated from autologous CD34+ progenitor cells, and 2) existing APCs (e.g., B cells, monocytes) from PBMCs (Fig. 5*A*). Following 10 d of priming and expansion, reactive T cells were isolated by fluorescence-activated cell sorting (FACS) based on either a surface activation marker (CD137) or intracellular markers (IFNγ and TNFα) using a previously published CLInt-seq protocol (39–41). 10X single-cell V(D)J sequencing was performed to recover paired TCR sequences. PBMCs from nine healthy individuals were screened. Five healthy donors showed T cell responses when stimulated by IRIS-predicted epitope pool by either CLInt-seq (Fig. 5*B*) or CD137 (Fig. 5*C*).

Isolated candidate TCRs were tested in a Jurkat-NFAT-GFP reporter system for rapid functional screening and cognate epitope deconvolution. NFAT-binding motifs followed by a GFP expression sequence were introduced in Jurkat cells coexpressing CD8. Upon T cell activation, GFP expression would be induced by transcription factor NFAT (42). TCRα/β pairs isolated from sequencing were synthesized and reconstructed into a single fragment by the F2Aopt linker in the pMAX plasmid to ensure equal copies of both alpha and beta chains (Fig. 5*D*). Plasmids were then transfected into Jurkat-NFAT-GFP cells via electroporation. For higher throughput, we adopted a pooling strategy to deconvolute reactive pools to a single peptide (Fig. 5*E* and *SI Appendix*, *Supplementary Materials and Methods*). A total of 21 TCRs derived from the five healthy donors recognized the peptide pool (Dataset S3b). Of these, seven TCRs reacted to a single IRIS-predicted epitope in Jurkat-NFAT-GFP cells.

TCRs that showed a response in the Jurkat-NFAT-GFP screening were then selected for engineering into healthy donor PBMCs via retroviral transduction to confirm functional reactivity and cytotoxicity (*SI Appendix*, *Supplementary Materials and Methods*). When expressed in PBMCs, the seven TCRs recognized four exogenously added IRIS-predicted epitopes, as measured by the production of IFNγ (*SI Appendix*, Fig. S6*A* and Dataset S3c). One TCR (JPTCR_47) showed similar reactivity toward its target as compared to the clinically tested F5 TCR toward its cognate antigen (MLANA, also known as MART1) (43), as measured by peptide serial-dilution assays (*SI Appendix*, Fig. S6*B*).

To test if the isolated TCRs could recognize processed epitopes on HLA-A*02:01, truncated isoforms for five TCR-epitope pairs were introduced into target K562 cells that coexpressed A*02:01 (K562-A2). IFNγ ELISA results confirmed the reactivity of JPTCR_238 in PBMCs when cocultured with both truncated and full-length cytoplasmic linker associated protein 1 (CLASP1) isoforms containing the IRIS-predicted epitope of interest (Fig. 5*F*). Cytotoxicity results measured by Incucyte live-cell analysis showed recognition and killing of target cells by JPTCR_238 (Fig. 5*G*). In contrast, cells under all three negative control conditions (NGFR, F5, untransduced) were growing during the entire Incucyte live-cell analysis, with minor differences in their growth rates. Taken together, our data provide experimental evidence that antigen-reactive TCRs can target IRIS-predicted AS-derived epitopes with high potency and specificity.

## Discussion

We introduce IRIS, a computational framework that leverages large-scale RNA-seq data for the discovery of AS-derived TAs. We demonstrated the utility of IRIS with a proof-of-concept analysis using paired RNA-seq and immunopeptidomics data of three human cell lines. We performed an in-depth analysis of a metastatic and highly lethal prostate cancer, NEPC, to evaluate the ability of IRIS to discover AS-derived immunotherapy targets in tumor specimens. The NEPC-specific AS events that we identified were highly enriched for inclusion of microexons, pointing to a distinct program of splicing dysregulation in this aggressive disease (37). By employing in vitro T cell priming and subsequent single-cell TCRα/β sequencing based on T cell activation markers, we established that IRIS-predicted NEPC epitopes could be recognized by T cells. These data provide experimental evidence for antigen-reactive TCR efficacy against AS-derived epitopes.

IRIS represents a systematic and generalizable strategy for exploiting AS as a source of cancer immunotherapy targets. By performing multiple types of in silico screening tests against a large-scale reference database of AS profiles of tumor and normal tissues (IRIS DB), IRIS can identify and prioritize AS-derived targets with varying degrees of tumor association and specificity. Importantly, to prioritize tumor-specific targets, IRIS incorporates a SJ count-based tumor-specificity screen to test the presence-absence of any given SJ in tumor and normal tissue samples, allowing detection of AS-derived targets with “neoantigen-like” tumor specificity. Additionally, by examining RNA-seq data from the same cancer type through the tumor-recurrence screen, IRIS can discover TAs shared among patients from multiple cohorts. Collectively, IRIS’s ability to perform comprehensive screening tests along with its associated data resource (IRIS DB) provides a significant advantage over existing target discovery pipelines (16, 17, 22, 23) and facilitates selection of AS-derived targets with low off-tumor toxicity and broad clinical applicability.

We assessed and validated IRIS-predicted epitopes via independent approaches. We initially performed a proof-of-concept analysis integrating RNA-seq and immunopeptidomics data. We confirmed the presence of IRIS-predicted epitopes in the HLA-I immunopeptidome of multiple human cell lines (Fig. 2). As expected, predicted AS-derived epitopes from transcripts with higher expression levels and corresponding to peptides with stronger predicted HLA binding affinities were more likely to be detected in immunopeptidomics data (Fig. 2*E*). We then applied IRIS for TCR target discovery for NEPC, a highly lethal prostate cancer with no effective long-term treatments or targeted therapies. IRIS identified 2,939 NEPC-associated SE events, among which 87 were identified as NEPC-specific. NEPC-specific SE events were significantly enriched for NEPC-specific inclusion of microexons (Fig. 3*D*), which are known to be up-regulated in neuronal cell lineages and may underlie the neuroendocrine transformation of prostate cancer cells in NEPC (44). We noted that elevated expression of the splicing factor SRRM4 in NEPC correlated with these results (Fig. 3*F*). We experimentally isolated seven unique TCRs specifically recognizing four unique IRIS-predicted epitopes. We tested one TCR that showed efficient killing of target cells expressing the target protein (Fig. 5*G*). Our work demonstrates that AS-derived epitopes predicted by IRIS can be processed and presented on HLA-I and recognized by a cognate TCR discovered from healthy donor PBMCs. Further applications of the workflow shown in this work should eventually result in new targets and therapeutic TCRs for many types of cancer. Combining advanced experimental tools, such as using the artificial thymic organoid (ATO) system (45, 46) as an alternative source of T cells, could benefit target validation and TCR discovery.

We note that user-provided RNA-seq data may have different read lengths from the RNA-seq data in the IRIS DB (e.g., GTEx and TCGA). Although rMATS-turbo accounts for RNA-seq read length in estimating PSI values and the effect of read length on AS quantification and consequently target discovery is minor (28), we cannot rule out the possibility that certain IRIS-predicted targets may be sensitive to variation in RNA-seq read length. It is possible to identify and flag such AS events by 1) trimming RNA-seq reads from user-provided RNA-seq data to match the read lengths in the IRIS DB, and then 2) assessing changes in the estimated PSI values.

The current IRIS platform has several limitations. IRIS uses short-read RNA-seq data for AS analysis. Although short-read RNA-seq has been the standard technology for transcriptome analysis, it has an inherent limitation for inferring full-length transcript isoforms and their corresponding protein products (47). Because short-read RNA-seq only examines fragments of full-length transcripts, protein products that correspond to the identified AS events often cannot be reliably inferred, particularly for events involving complex AS patterns or novel unannotated SJs. Currently, target discovery in IRIS is limited to peptides encoded by SJs corresponding to five basic types of binary AS patterns (Fig. 1); thus, a considerable number of potential epitopes, including those derived from complex AS events, are not considered. The long-read RNA-seq technology, which is ideally suited for analyzing full-length transcript and protein isoforms, may overcome the limitation of short-read RNA-seq and enable a more comprehensive and robust approach for TA discovery (14, 48–50). Additionally, the current IRIS platform and its associated IRIS DB are based on bulk RNA-seq data and lack single-cell and spatial resolution. The clonality, heterogeneity, and plasticity of AS-derived immunotherapy targets represent important biological features that may affect therapy efficacy and outcome (14). Although IRIS is designed to discover AS-derived targets with significantly higher expression in tumor tissues over normal tissues, including those with “neoantigen-like” tumor-specific expression, we currently do not know the clonality of these AS-derived targets. Whether all or a subset of cancer cells in a given tumor express an AS-derived target of interest remains an open question. Going forward, isoform-resolved single-cell or spatial RNA-seq datasets may enhance the resolution of transcriptome profiles in IRIS, by providing cell type-specific or spatial information (14). Of note, long-read single-cell RNA-seq has emerged as a powerful technology for transcript isoform analysis in single cells (51–53). We plan to incorporate long-read RNA-seq data, at both bulk and single-cell levels, in our future development of IRIS.

In summary, IRIS represents a big-data informed computational platform to discover AS-derived cancer immunotherapy targets. In this study, we focused on the application and validation of IRIS for discovering TCR targets. Our results provide experimental evidence for the immunogenicity of AS-derived epitopes and suggest their potential for therapy development. The IRIS software can be downloaded from https://github.com/Xinglab/IRIS.

## Materials and Methods

### IRIS Module for RNA-seq Data Processing.

IRIS accepts raw short-read RNA-seq data (FASTQ files) and/or tab-delimited files of AS events quantified by rMATS-turbo (26, 28) as input data. For raw RNA-seq data, IRIS provides a stand-alone pipeline that aligns RNA-seq reads to the reference human genome, quantifies gene expression, and characterizes AS events. In this work, the IRIS RNA-seq data processing module used the reference human genome hg19 and STAR 2.6.1d (54) under the two-pass mode for RNA-seq read alignment. Gene expression and AS events were quantified using Cufflinks v2.2.1 (55) and rMATS v4.1.0 under default parameters, respectively, based on the GENCODE (V26) (56) gene annotation. To quantify AS events, IRIS extracts PSI values (29) for all AS events in the rMATS-turbo output file and read counts for all SJs in the RNA-seq alignment file. To remove low-confidence PSI estimates, AS events with low RNA-seq coverage, defined as events with an average read count of less than 10 for the sum of all corresponding SJs in a given sample or sample group (e.g., tumor or normal tissue type), are masked as having missing values in the output file of IRIS-characterized AS events. This pipeline discovers and quantifies all major types of AS events, including SE, A5SS, A3SS, and RI events. AS events characterized by IRIS may involve either annotated or novel SJs of annotated splice sites. The option to discover and quantify AS events involving novel splice sites is also available through the --novelSS option of rMATS-turbo. This pipeline was uniformly applied to all RNA-seq datasets in this work, including the reference datasets of tumor and normal tissue samples (TCGA, GTEx) used for generating the IRIS DB.

### IRIS DB: A Reference Database of AS Profiles across Tumor and Normal Tissue Samples.

IRIS utilizes IRIS DB, a reference database of AS profiles across a diverse panel of tumor and normal tissue samples, to identify AS events with varying degrees of tumor association and specificity. Specifically, 9,932 tumor tissue samples from TCGA (16, 25) representing 33 tumor types were uniformly processed as described above. In addition, 9,024 normal tissue samples from GTEx (V7) (57), representing 51 normal tissue types of 30 histological sites, were also processed. Cell line samples from GTEx were excluded from the IRIS DB. The IRIS DB contains ratio-based (PSI) (29) and count-based (SJ read count) quantification of all AS events detected in TCGA and GTEx. A summary of the IRIS DB is provided in *SI Appendix*, Table S1. PSI values and SJ read counts stored in the IRIS DB are indexed using their genomic coordinates, gene identifiers, and gene symbols as keys. The IRIS DB, together with the IRIS functions to retrieve user-selected tumor and normal tissue types from the IRIS DB to create custom reference panels, are made available as stand-alone resources. In addition, IRIS provides functions for users to build and index their own RNA-seq datasets into custom reference panels.

### IRIS Module for In Silico Screening.

IRIS performs in silico screening to identify AS events of varying degrees of tumor association and specificity, by comparing user-provided RNA-seq data of tumor samples to a reference panel of user-specified tumor and normal tissue types selected from the IRIS DB. IRIS’s in silico screening module provides three types of screening tests, including a tumor-association screen, a tumor-specificity screen, and a tumor-recurrence screen.

The default tumor-association screen performs a differential AS analysis between tumor and normal tissues based on the PSI metric. For each AS event, IRIS compares its PSI values between user-provided RNA-seq data of tumor samples and a given normal tissue type in the reference panel selected from the IRIS DB. IRIS reports a differential AS event based on various user-defined criteria, such as the *P*-value, the change of PSI value (delta PSI), and the fold-change (FC) of the tumor-enriched isoform (see below for a detailed definition). Specifically, to define a differential AS event, IRIS sets two default requirements: 1) a significant *P*-value from a statistical test (default: two-sided *t* test *P* < 0.01, unequal variance allowed), and 2) a threshold of average PSI value difference [default: abs(ΔPSI) > 0.05]. For each AS event, IRIS defines its degree of tumor association as the number of normal tissue types compared to which the tumor samples have consistently and significantly higher or lower PSI values. An AS event is defined as tumor-associated if its degree of tumor association reaches a user-defined threshold. In this work, we selected 11 normal tissue types from the IRIS DB into the reference panel, and the threshold for the degree of tumor association is set as 8. For each AS event defined as tumor associated, IRIS defines the tumor-enriched isoform as the isoform that is more abundant in the tumor samples compared to the normal tissue panel. The FC of the tumor-enriched isoform is calculated as the fold-change of the proportion of the tumor-enriched isoform in tumor tissues over the average proportion of the tumor-enriched isoform in all normal tissue types of the normal tissue panel. This metric can be used to evaluate and visualize predicted targets (Fig. 4).

IRIS also provides an optional, secondary tumor-association screen based on normalized SJ read counts in the unit of counts per million (CPM). For a given SJ in a given sample, the CPM value is the raw SJ read count multiplied by a normalization factor, 10^6^/R, where R is the total count of all mapped RNA-seq reads in the sample. This screening test directly compares the expression level of a given SJ between tumor and normal tissues. A one-sided *t* test is used to assess if a given SJ is expressed at a significantly higher level in tumor samples as compared to normal tissue samples. Similar to the PSI-based screening test described above, a one-sided *t* test *P* < 0.01 is required to call the SJ CPM-based screening test significant against a given normal tissue type. For each SJ, the degree of tumor association is similarly defined as the number of normal tissue types compared to which the tumor samples have significantly higher CPM values.

The tumor-specificity screen tests and compares the presence-absence of a given SJ between tumor and normal tissues. Specifically, for each sample group (e.g., user-provided RNA-seq data of tumor samples, or a reference normal tissue type in the IRIS DB), IRIS calculates the percentage of samples expressing a given SJ at or above a user-defined read count threshold. The default threshold is set as 5 for tumor samples and 2 for normal tissue samples. IRIS then performs a one-sided Fisher Exact test to determine if a given SJ is expressed in a significantly higher percentage of tumor samples than in normal tissue samples of a given normal tissue type (default: *P* < 1 × 10^−6^). IRIS defines a SJ as tumor-specific, if the number of significant tests against the normal tissue panel reaches a user-defined threshold (8 out of 11 normal tissue types tested in this work). Finally, IRIS reports a tumor-associated AS event as tumor-specific if all SJ(s) of its corresponding tumor-enriched isoform are tumor-specific as defined by the tumor-specificity screen.

Finally, IRIS provides a tumor-recurrence screen, to identify AS events that are recurrent (shared) among independent cohorts of the similar tumor type. This screening test is described in detail in *SI Appendix*, *Supplementary Materials and Methods*.

### IRIS Module for Target Prediction.

To obtain peptide sequences of AS-derived tumor-enriched isoforms, IRIS translates SJ sequences into peptide sequences using annotated reading frames from the UniProtKB database (31). Specifically, for the upstream exon of a given SJ in a given AS event, IRIS identifies its reading frame based on the corresponding protein product annotated in UniProtKB. The identified reading frame of the upstream exon is then used for translating the SJ of interest into its corresponding peptide. For each AS event, the SJ peptide of the tumor-enriched isoform is compared to the SJ peptide of the normal-enriched isoform to ensure that they produce distinct peptide sequences. By default, SJ peptides are 21 amino acids in length and centered at the splice sites, but the actual lengths may vary depending on the exon lengths.

For TCR target prediction, IRIS employs seq2HLA (58), which uses RNA-seq data to determine HLA class I alleles for each tumor sample. IRIS then uses the IEDB API (30) predictors to obtain putative HLA binding affinities of candidate peptides. The IEDB “recommended” mode runs multiple tools to generate predictions of binding affinity, which IRIS summarizes as a median IC_50_ value. By default, a threshold of median(IC_50_) < 500 nM defines a positive prediction for an AS-derived TCR target. For CAR-T target prediction, detailed descriptions are available in *SI Appendix*, *Supplementary Materials and Methods* and Fig. S2.

### Proteotranscriptomics Data Integration for MS Validation.

IRIS includes an optional proteotranscriptomics data integration function that incorporates various types of MS data, such as whole-cell proteomics, surfaceomics, and immunopeptidomics data, to validate RNA-seq-based target discovery at the protein level (Fig. 2). Specifically, sequences of AS-derived peptides are added to canonical and isoform sequences of the reference human proteome (downloaded from UniProtKB in September 2018). For immunopeptidomics data, fragment MS spectra are searched against the RNA-seq-augmented custom proteome library with no enzyme specificity using MSGF+ (59) with the search length limited to 7 to 15 amino acids. The target-decoy approach is employed to control the FDR or “QValue” at 5%.

### IRIS Analysis of Immunopeptidomics Data.

Data sources for the IRIS immunopeptidomics data analysis are reported in Data, Materials, and Software Availability. RNA-seq data of normal (B-LCL-S1 and B-LCL-S2) and cancer (JeKo-1) cell lines were analyzed by IRIS as described above, with minor modifications. Specifically, AS events identified by the IRIS RNA-seq data processing module (with STAR v2.5.3a and rMATS v4.0.2) were not subjected to the in silico screening module, but instead were directly used for the MS search. For MSGF+, FDR was set at 5% for Fig. 2 *B* and *E*. For the comparison of AS-derived peptides with high and low predicted HLA binding affinities (Fig. 2*D*), a set of low-affinity peptides was created by randomly selecting peptides with median(IC_50_) ≥ 500 nM to the same number of high-affinity peptides [median(IC_50_) < 500 nM]. The transcript expression level was approximated by taking the product of the gene expression level (FPKM) of the AS event’s corresponding gene and the PSI or 1-PSI value for exon inclusion or skipping SJ(s), respectively.

### IRIS Analysis of 23 NEPC Samples.

Database accession numbers for the NEPC, CRPC, and PRAD RNA-seq data are reported in Data, Materials, and Software Availability. In total, 23 NEPC samples were included in this study. Splicing factor (36) gene expression levels were quantified by FeatureCounts v2.0.1 (60), followed by DESeq2 v1.26.0 (61) normalization.

Default screening parameters were used for the IRIS analysis of NEPC, with minor modifications. Default parameters were used to perform both tumor-association and tumor-specificity screens. For the reference panel, the normal tissue panel was comprised of 11 normal tissue types selected from the IRIS DB, including heart, blood, lung, liver, brain, nerve, muscle, spleen, thyroid, skin, and kidney. We did not have RNA-seq data for a second cohort of NEPC samples to perform the tumor-recurrence screen. For each test, the minimum number of NEPC samples with nonmissing values was required to be three, and the equal variance option was enabled for the *t* test. For TCR target discovery, two common HLA types, HLA-A*02:01 and HLA-A*03:01, were used for prediction. Default parameters for TCR target prediction were used.

### Target Selection for Experimental Validation.

From the pool of 1,651 IRIS-predicted tumor-associated TCR epitopes, a subset of candidate epitopes was selected for experimental validation. We applied three additional criteria: 1) restriction to HLA-A*02:01; 2) high FC of the tumor-enriched isoform (FC ≥ 2); and 3) high gene expression level (average FPKM ≥ 20). We obtained 164 candidate epitopes that met these criteria. From these epitopes, and additional 52 epitopes derived from NEPC-specific SJs identified by the tumor-specificity screen, we selected 76 epitopes to test for immunogenicity and T cell recognition (Dataset S3a).

### Additional Information of Computational and Experimental Procedures.

Additional technical information of IRIS (e.g., additional features of IRIS screening and prediction modules, target annotations) as well as experimental procedures for target validation and TCR analysis [e.g., cell culture, T cell priming and activation, single-cell V(D)J sequencing, TCR screening, and functional analyses] are described in *SI Appendix*, *Supplementary Materials and Methods*.

## Supplementary Material

Appendix 01 (PDF)

Dataset S01 (XLSX)

Dataset S02 (XLSX)

Dataset S03 (XLSX)

## Data Availability

The IRIS source code is accessible on GitHub at https://github.com/Xinglab/IRIS. The IRIS Explorer for exploring and visualizing IRIS results is available at https://xingshiny2.research.chop.edu/shiny/IRIS/. The RNA-seq data of 23 NEPC samples were retrieved from a Beltran et al. study (accession no. phs000909) (62) and a Stand Up To Cancer (SU2C) study (accession no. phs000915) (63). FASTQ files were downloaded from the database of Genotypes and Phenotypes (dbGAP). RNA-seq data of CRPC samples were obtained from the Beltran et al. study (accession no. phs000909) (62), the SU2C study (accession no. phs000915) (63), and a Robinson et al. study (accession no. phs000673) (64). RNA-seq data of PRAD samples were downloaded as part of the TCGA data for the IRIS DB from GDC via gdc-client (65). RNA-seq data used to construct the IRIS DB are available from TCGA (https://portal.gdc.cancer.gov/legacy-archive/) (25) and GTEx (https://gtexportal.org/) (57). For the IRIS proteotranscriptomics analysis, matching RNA-seq data and MS immunopeptidomics data of B-LCL-S1 and B-LCL-S2 cell lines were retrieved from Laumont et al. (GEO: GSM1641206, GSM1641207, and PRIDE: PXD001898) (32). RNA-seq data of the JeKo-1 lymphoma cell line were obtained from the Cancer Cell Line Encyclopedia (66) via the NCI Genomic Data Commons (https://portal.gdc.cancer.gov/legacy-archive/). Corresponding MS immunopeptidomics data of JeKo-1 were retrieved from Khodadoust et al. (PRIDE: PXD004746) (33).
