# Supplementary material for: IRIS: Discovery of cancer immunotherapy targets arising from pre-mRNA alternative splicing
Source: Proc Natl Acad Sci U S A. 2023 May 16;120(21):e2221116120. doi: 10.1073/pnas.2221116120 (PMC10214192; doi:10.1073/pnas.2221116120)
Supplement: Supplementary file 1 — Appendix 01 (PDF) [file pnas.2221116120.sapp.pdf]

PNAS

Supplementary Information for

IRIS: discovery of cancer immunotherapy targets arising from pre-mRNA alternative splicing

Yang Pan, John W. Phillips, Beatrice D. Zhang, Miyako Noguchi, Eric Kutschera, Jami McLaughlin, Pavlo A. Nesterenko, Zhiyuan Mao, Nathanael J. Bangayan, Robert Wang, Wendy Tran, Harry T. Yang, Yuanyuan Wang, Yang Xu, Matthew B. Obusan, Donghui Cheng, Alex H. Lee, Kathryn E. Kadash-Edmondson, Ameya Champhekar, Cristina Puig-Saus, Antoni Ribas, Robert M. Prins, Christopher S. Seet, Gay M. Crooks, Owen N. Witte*, and Yi Xing*

*These authors jointly supervised this work: Yi Xing, Owen N. Witte
Email: xingyi@chop.edu; owenwitte@mednet.ucla.edu

This PDF file includes:

Figures S1 to S5
Table S1
Legends for Datasets S1 to S3
Supplementary Materials and Methods
Supplementary References

Other supplementary materials for this manuscript include the following:

Datasets S1 to S3

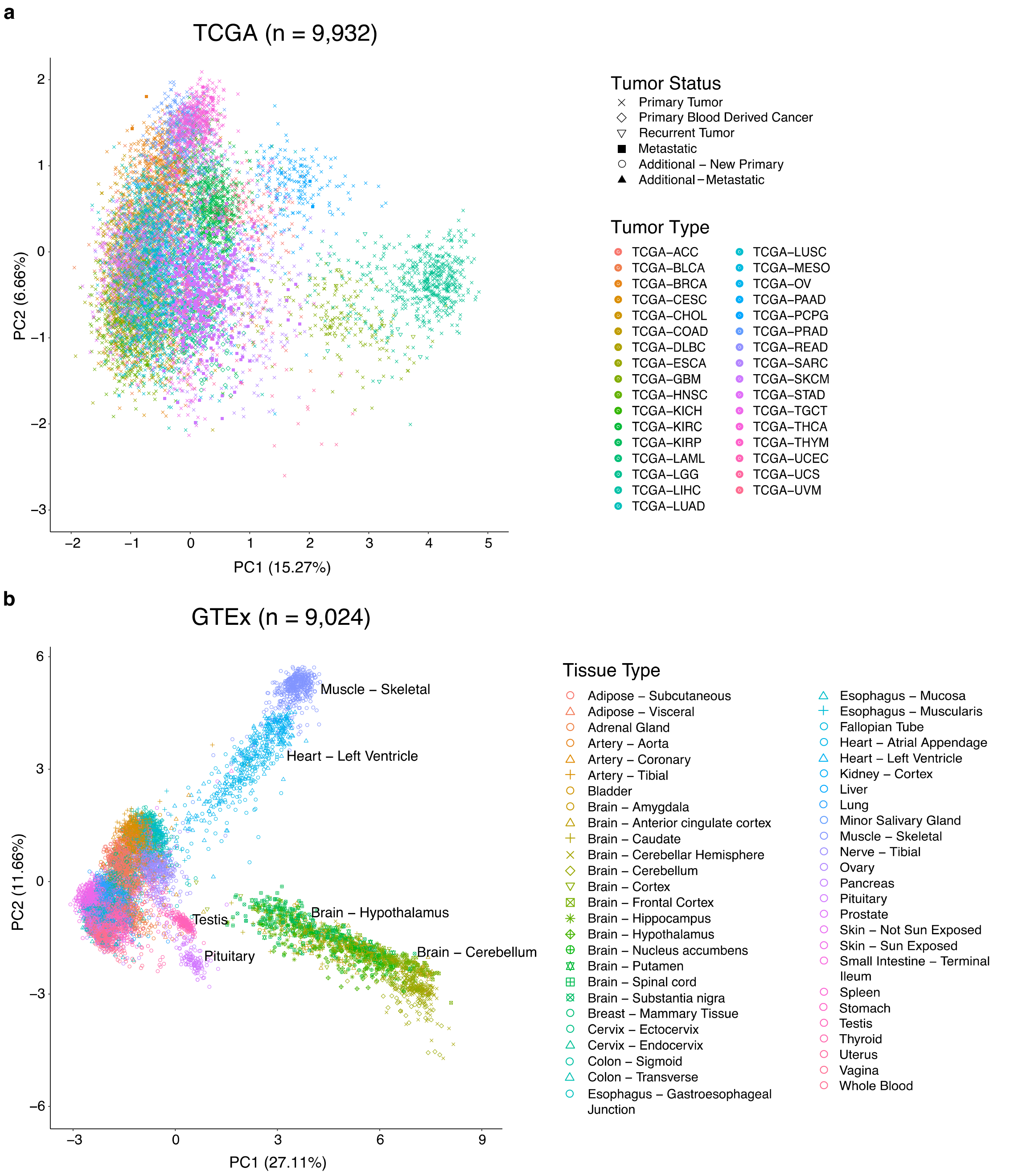

Figure S1. IRIS DB: A reference database of alternative splicing (AS) profiles across tumor and normal tissue samples. a, Percent-spliced-in (PSI)-based principal component analysis (PCA) of RNA-seq data of 9,932 samples from 33 tumor types from the TCGA consortium. **b,** PSI-based PCA of RNA-seq data of 9,024 samples from 51 normal tissue types of 30 histological sites from the GTEx consortium.

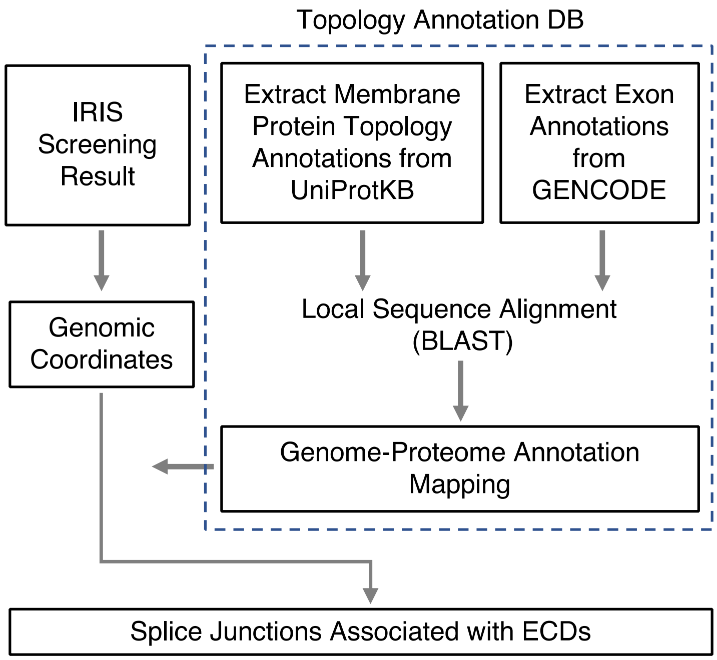

Figure S2. CAR-T target prediction by IRIS. Computational workflow for annotating protein extracellular domain (ECD)-associated AS events for discovering chimeric antigen receptor T-cell (CAR-T) targets.

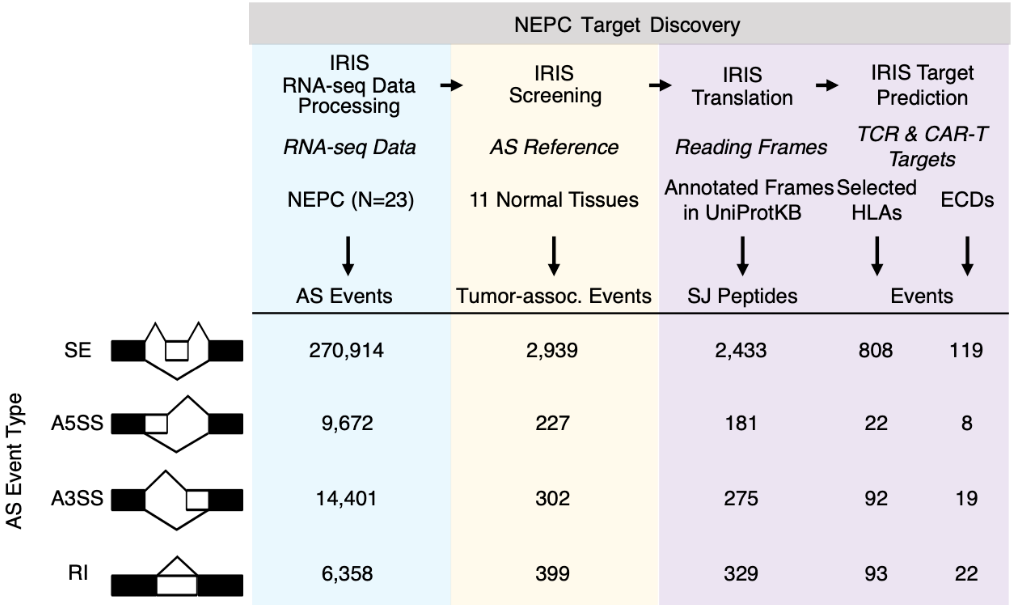

Selected HLA Types: HLA-A02:01, HLA-A03:01
ECD information is derived from UniProtKB annotations

Figure S3. IRIS discovery of AS-derived targets for NEPC arising from four types of AS events. Stepwise results of IRIS to identify AS-derived cancer immunotherapy targets from 23 neuroendocrine prostate cancer (NEPC) samples. Skipped exon (SE), alternative 5' splice sites (A5SS), alternative 3' splice sites (A3SS), and retained intron (RI) events identified by the IRIS RNA-seq data processing module (blue) were screened against 11 normal tissue types from the IRIS DB (yellow) to identify tumor-associated events and predict corresponding T-cell receptor (TCR) and CAR-T targets (purple). ECD, extracellular domain; HLA, human leukocyte antigen; SJ, splice junction.

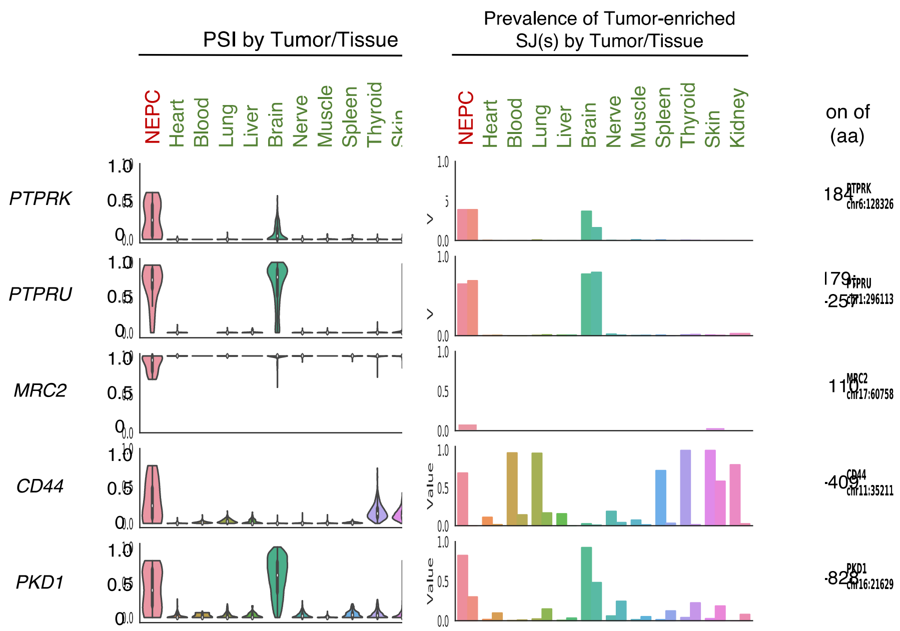

Figure S4. Representative examples of 5 IRIS-predicted CAR-T targets for NEPC. IRIS-predicted CAR-T targets are visualized by IRIS in paired violin and bar plots. Each row shows one IRIS-predicted CAR-T target. Violin plots show the PSI values of each target in NEPC and the normal tissue panel. Bar plots show the fraction of samples expressing the SJ(s) of the tumor-enriched isoform in NEPC and the normal tissue panel. If the tumor-enriched isoform is the exon inclusion isoform, the bar plot displays the upstream and downstream inclusion SJ as two bars. If the tumor-enriched isoform is the exon skipping isoform, the bar plot displays the skipping SJ as one bar. Positions of ECDs in amino acid (aa) sequences of the corresponding UniProtKB canonical proteins are shown for individual CAR-T targets. ECD, extracellular domain; PSI, percent spliced in; SJ, splice junction.

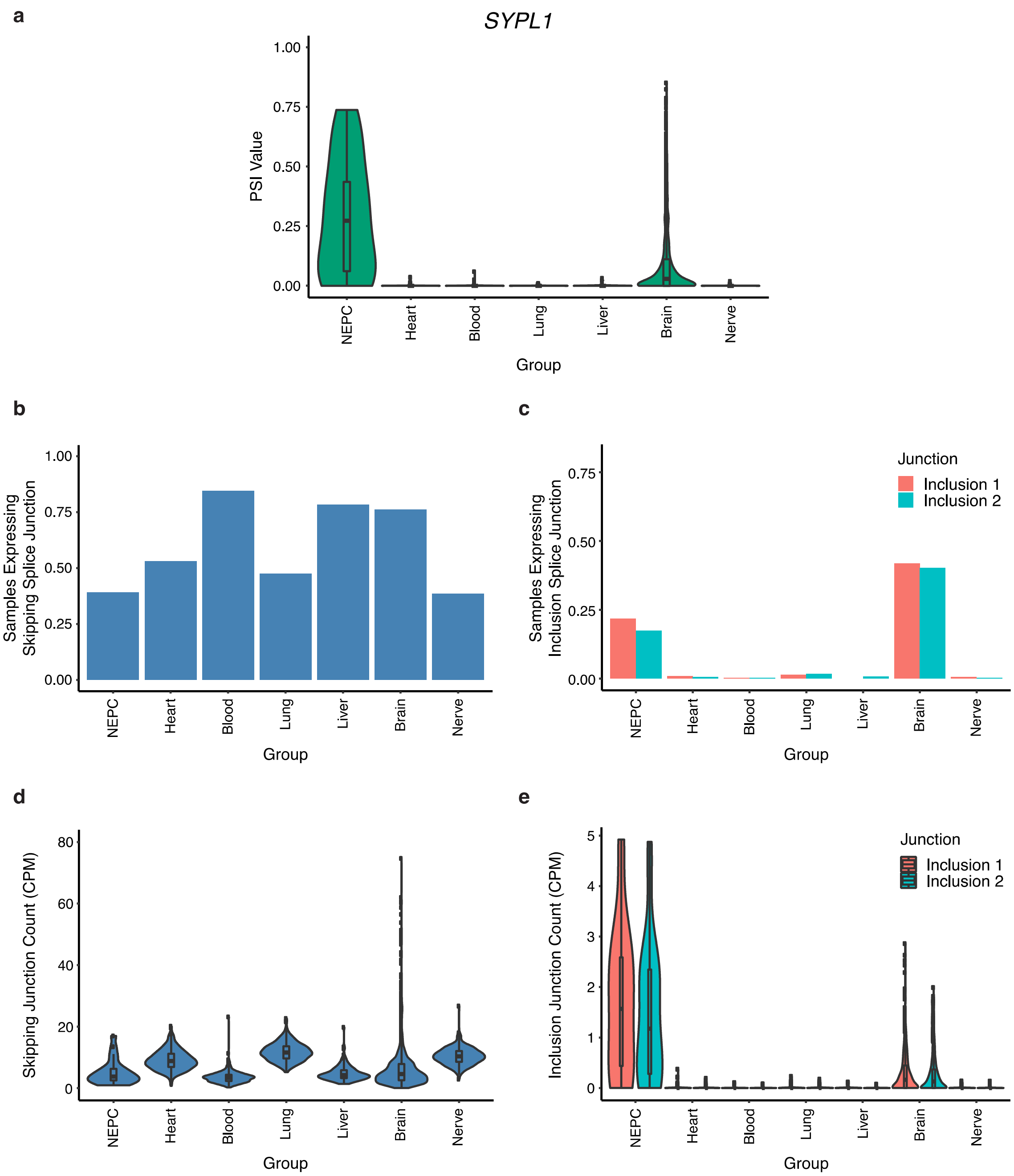

Figure S5. IRIS Explorer: a web-based tool to explore and visualize IRIS results. Shown are various visualizations generated by the web-based tool, IRIS Explorer, using an AS event in SYPL1 as an example. For this AS event, we used IRIS Explorer to generate five visualizations using data across NEPC and six selected normal tissue types from the IRIS DB (heart, blood, lung, liver, brain, and nerve). **a**, Violin plots show the PSI values in NEPC and the normal tissue panel. **b**, Bar plots show the fraction of samples expressing the skipping SJ in NEPC and the normal tissue panel. **c**, Bar plots show the fraction of samples expressing the inclusion SJs in NEPC and the normal tissue panel. The upstream and downstream inclusion SJs are displayed as two bars. **d**, Violin plots show the SJ count (in CPM) of the skipping SJ in NEPC and the normal tissue panel. **e**, Violin plots show the SJ count (in CPM) of the inclusion SJs in NEPC and

the normal tissue panel. The upstream and downstream inclusion SJs are displayed as two violin plots. CPM, counts per million; SJ, splice junction.

a

b

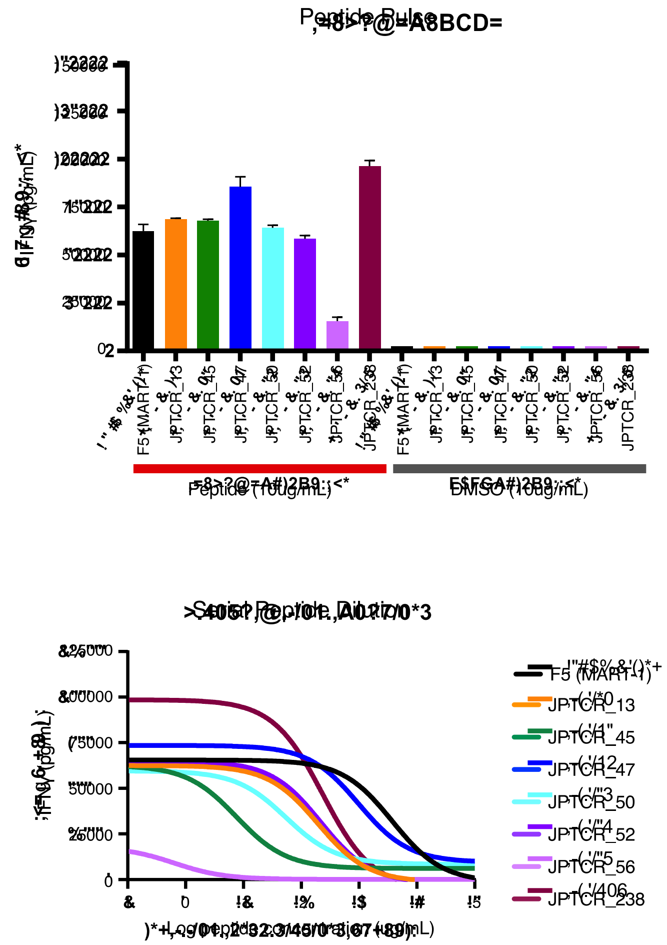

Figure S6. IFNγ ELISA of TCR-transduced PBMCs co-cultured with K562-A2 and exogenously added peptides. a, IFNγ ELISA of TCR-transduced peripheral blood mononuclear cells (PBMCs) co-cultured with K562 cells expressing HLA-A*02:01 (K562-A2) antigen-presenting cells (APCs) pulsed with a single cognate peptide. Error bars represent standard deviation (n=3). **b**, IFNγ ELISA of TCR-transduced PBMCs co-cultured with K562-A2 APCs in a dilution series of cognate peptide.

Table S1. Summary of IRIS DB.

TCGA Summary

Tumor Type	Sample Number	SE	A5SS	A3SS	RI	AS Events*	SJs
**ACC**	79	120,645	6,254	9,730	5,484	142,113	1,180,943
**BLCA**	414	257,419	9,422	13,998	6,126	286,965	3,982,733
**BRCA**	1,105	371,719	11,332	16,449	6,310	405,810	7,504,829
**CESC**	306	242,113	9,145	13,447	6,048	270,753	3,525,799
**CHOL**	36	100,375	5,855	9,211	5,433	120,874	891,934
**COAD**	300	210,640	8,527	12,503	5,916	237,586	2,986,662
**DLBC**	48	98,450	5,829	9,005	5,236	118,520	1,053,528
**ESCA**	185	337,750	10,401	15,422	6,287	369,860	9,946,490
**GBM**	170	254,675	8,797	13,119	5,878	282,469	2,988,768
**HNSC**	522	290,258	9,631	14,224	6,038	320,151	4,969,613
**KICH**	66	124,713	6,302	9,776	5,363	146,154	1,304,627
**KIRC**	542	267,169	9,487	14,083	6,089	296,828	4,717,747
**KIRP**	291	193,373	7,989	12,181	5,873	219,416	2,922,051
**LAML**	179	191,636	7,681	11,279	5,619	216,215	5,327,364
**LGG**	534	285,850	9,445	14,063	5,964	315,322	4,971,783
**LIHC**	374	198,980	8,039	12,157	5,862	225,038	3,210,316
**LUAD**	541	276,002	9,779	14,506	6,156	306,443	4,768,990
**LUSC**	502	312,798	10,270	15,044	6,230	344,342	5,321,761
**MESO**	87	144,046	6,779	10,438	5,614	166,877	1,627,239
**OV**	430	399,702	11,859	17,292	6,387	435,240	19,933,273
**PAAD**	179	183,062	7,693	11,680	5,834	208,269	2,293,210
**PCPG**	184	164,172	7,251	10,977	5,639	188,039	2,285,776
**PRAD**	502	220,607	8,616	12,873	5,904	248,000	4,178,440
**READ**	95	142,669	6,815	10,372	5,532	165,388	1,551,651
**SARC**	263	235,170	8,682	13,061	5,968	262,881	3,076,820
**SKCM**	472	270,894	9,449	14,081	6,047	300,471	4,425,773
**STAD**	416	379,192	11,315	16,748	6,363	413,618	14,208,112
**TGCT**	156	181,217	7,961	12,203	5,937	207,318	2,248,877
**THCA**	513	235,692	8,703	13,132	5,869	263,396	4,266,263
**THYM**	120	152,603	7,279	11,241	5,714	176,837	1,878,900
**UCEC**	184	183,138	8,054	12,114	5,937	209,243	2,353,720
**UCS**	57	134,361	6,769	10,409	5,695	157,234	1,252,752
**UVM**	80	100,513	5,827	9,086	5,202	120,628	1,112,820

*AS events are rMATS-detected AS events with splice junction read coverage ≥ 10.
Abbreviations: TCGA, The Cancer Genome Atlas; SE, skipped exon; A5SS, alternative 5' splice sites; A3SS, alternative 3' splice sites; RI, retained intron; AS, alternative splicing; SJ, splice junction.

GTEx Summary

Tissue Type	Sample Number	SE	A5SS	A3SS	RI	AS Events*	SJs
**Adipose Tissue**	609	769,245	16,414	23,190	6,380	815,229	5,150,709
**Adrenal Gland**	160	666,677	14,572	20,842	6,035	708,126	2,122,719
**Bladder**	10	599,282	12,705	18,154	5,668	635,809	600,227
**Blood**	440	682,594	14,624	21,130	6,023	724,371	3,245,554
**Blood Vessel**	743	761,330	16,164	22,833	6,267	806,594	5,643,637
**Brain**	1,392	824,639	17,066	24,279	6,491	872,475	8,668,736
**Breast**	218	731,447	15,912	22,478	6,293	776,130	2,929,950
**Cervix**	11	617,162	13,239	18,825	5,795	655,021	638,697
**Colon**	379	721,064	15640	22,122	6,289	765,115	3,755,536
**Esophagus**	797	754,922	16,354	23,056	6,381	800,713	5,977,698
**Fallopian Tube**	7	610,808	13,067	18,840	5,865	648,580	557,802
**Heart**	476	701,917	15,130	21,354	6,137	744,538	3,569,386
**Kidney**	36	654,464	14,300	20,453	6,121	695,338	1,070,133
**Liver**	138	627,030	13,808	19,847	5,975	666,660	1,856,557
**Lung**	351	761,355	16,336	23,092	6,349	807,132	4,256,257
**Muscle**	464	705,864	15,015	21,128	6,033	748,040	3,516,485
**Nerve**	338	742,640	16,036	22,851	6,299	787,826	3,753,055
**Ovary**	112	685,042	14,976	21,229	6,141	727,388	2,023,161
**Pancreas**	192	649,198	14,345	20,453	6,050	690,046	1,983,912
**Pituitary**	130	714,640	15,469	22,166	6,326	758,601	2,335,388
**Prostate**	120	702,864	15328	21,901	6,286	746,379	2,045,898
**Salivary Gland**	70	671,626	14,645	20,652	6,126	713,049	1,591,236
**Skin**	661	767,102	16,451	23,347	6,411	813,311	5,213,924
**Small Intestine**	103	687,188	15062	21,569	6,217	730,036	1,997,920
**Spleen**	118	644,182	14,446	20,806	6,091	685,525	2,011,201
**Stomach**	207	688,674	14967	21,217	6,181	731,039	2,361,544
**Testis**	201	767,536	16,601	23,516	6,517	814,170	6,681,759
**Thyroid**	345	744,351	16,174	22,994	6,338	789,857	4,031,882
**Uterus**	95	673,243	14,753	21,014	6,043	715,053	1,805,546
**Vagina**	101	689,681	15,085	21,243	6,179	732,188	1,906,189

*AS events are rMATS-detected AS events with splice junction read coverage ≥ 10.
Abbreviations: GTEx, Genotype-Tissue Expression; SE, skipped exon; A5SS, alternative 5' splice sites; A3SS, alternative 3' splice sites; RI, retained intron; AS, alternative splicing; SJ, splice junction.

Dataset S1 (separate file). IRIS proteo-transcriptomics analysis of alternative splicing (AS)-derived peptides in cell line immunopeptidomes. **a**. Summary of AS-derived epitopes in JeKo-1 cancer cell line. **b**. Summary of AS-derived epitopes in B-LCL-S1 normal cell line. **c**. Summary of AS-derived epitopes in B-LCL-S2 normal cell line.

Dataset S2 (separate file). IRIS analysis of neuroendocrine prostate cancer (NEPC). **a.** IRIS-identified NEPC-associated skipped exon (SE) events. **b.** Predicted HLA02:01-binding epitopes or HLA03:01-binding epitopes from IRIS-identified NEPC-associated skipped exon (SE) events. **c.** NEPC-associated skipped exon (SE) events located in annotated extracellular regions of cell-surface proteins. **d**, **e**, **f**. Results for alternative 5' splice sites (A5SS) events. **g**, **h**, **i**. Results for alternative 3' splice sites (A3SS) events. **j**, **k**, **l**. Results for retained intron (RI) events.

Dataset S3 (separate file). Selected IRIS-predicted NEPC epitopes for TCR isolation and characterization. **a**. Selected 76 IRIS-predicted NEPC epitopes for TCR isolation and characterization. **b**. Unique TCR clones recognizing the total IRIS-predicted epitope pool. **c**. Unique TCR clones specifically recognizing single IRIS-predicted epitopes.

Supplementary Materials and Methods

IRIS tumor-recurrence screen

IRIS's *in silico* screening module provides three distinct screening tests: a 'tumor-association screen', a 'tumor-specificity screen', and a 'tumor-recurrence screen', to identify alternative splicing (AS) events of varying degrees of tumor association and specificity. The first two screening tests are described in detail in the main manuscript. Here, we describe the tumor-recurrence screen, which allows IRIS to identify AS events that are recurrent (shared) among independent cohorts of the similar tumor type. Specifically, for tumor-associated AS events identified by the tumor-association screen, the tumor-recurrence screen compares tumor types of similar histology (*i.e.* independent tumor cohorts selected from the IRIS DB or provided by users) against a matched normal tissue type selected from the IRIS DB. To define a differential AS event in this test, IRIS sets two default requirements: 1) a significant p-value from a statistical test (default: one-sided t-test $p < 0.01$, unequal variance allowed) in the same direction as identified in the tumor-association screen, and 2) a threshold of average PSI value difference (default: abs(ΔPSI) > 0.05). IRIS defines an AS event as tumor-recurrent, if the number of significant tests for independent tumor cohorts against the matched normal tissue type reaches a user-defined threshold (*e.g.* 1 out of 2 independent tumor cohorts tested).

Additional criteria to evaluate IRIS-predicted targets

IRIS evaluates and visualizes predicted targets based on multiple criteria (**Fig. 4**). In addition to the three main criteria described in the main manuscript (degree of tumor association, FC of the tumor-enriched isoform between tumor and normal tissues, and gene expression level in tumor tissues), additional features are reported for predicted targets. The 'degree of tumor specificity' is the number of normal tissue types compared to which all SJ(s) of its corresponding tumor-enriched isoform are tumor-specific as defined by the SJ count-based tumor-specificity screen. The 'degree of tumor recurrence' is the number of independent tumor cohorts for which the AS event is defined as differential against a matched normal tissue type in the tumor-recurrence screen. The 'predicted HLA binding affinity' is the IEDB-predicted binding affinity for the epitope of interest. The 'mappability' is a measure of the SJ region's uniqueness or repetitiveness in the genome, based on the UCSC mappability track (1). The 'peptide uniqueness' indicates whether the SJ peptide sequence is unique within all annotated SJs and detected SJs in the analysis.

IRIS function for chimeric antigen receptor T-cell (CAR-T) target prediction

IRIS maps AS events to protein extracellular domains (ECDs) to discover potential CAR-T targets (**Fig. S2**). Specifically, IRIS collects and curates annotations of protein ECDs from UniProtKB (2). First, protein cellular localization information is retrieved from the UniProtKB database (flat file downloaded in April 2018). ECD information is retrieved by searching for the term 'extracellular' in topology annotation fields, including 'TOPO_DOM', 'TRANSMEM', and 'REGION', in the flat file. Next, BLAST (3) is used to map exons in the gene annotation (GENCODE V26) to proteins with topology annotations. Finally, the BLAST result is parsed to associate exons with protein ECDs. These curated annotations are used to identify SJs that overlap with protein ECDs as sources for potential AS-derived CAR-T targets.

Cell culture

Unless otherwise noted, all cell lines were obtained from ATCC (Manassas, VA). K562 cells expressing HLA-A*02:01 (K562-A2) were maintained in R10 media, which included RPMI 1640 (Thermo Fisher Scientific, CAT# 31800089) with 10% fetal bovine serum (FBS; Omega Scientific CAT# FB-11) and 4 mM L-glutamine (Thermo Fisher Scientific, CAT# BP379-100). Jurkat cells overexpressing the NFAT-GFP vector (Dr. David Baltimore, Caltech) and CD8 were maintained in R10 media. Peripheral blood mononuclear cells (PBMCs) from healthy HLA-A*02-01-positive donors were purchased from AllCells (Alameda, CA) and activated with CD3/CD28 Dynabeads (Gibco, Thermo Fisher Scientific, CAT# 11132D) in AIM-V media (Thermo Fisher Scientific, CAT# 12055091) supplemented with 5% heat-inactivated human AB serum (Omega Scientific, CAT# HS-20), 1X GlutaMAX (Gibco, Thermo Fisher Scientific, CAT# 35050-061), 55 μM β-mercaptoethanol (Sigma, CAT# M3148), 50 U/mL IL-2 (Peprotech, CAT# 200-02), and 1 ng/mL

IL-15 (Peprotech, CAT# 200-15). PBMCs were transduced with a retroviral vector encoding candidate TCRs as previously described (4). MS5-DLL1 cells were cultured in Dulbecco's Modified Eagle's Medium (DMEM; Thermo Fisher Scientific, CAT# 12100-061) supplemented with 10% FBS and 1% GlutaMAX.

Human granulocyte-colony stimulating factor (G-CSF)-mobilized peripheral blood was purchased commercially (HemaCare, Northridge, CA). Leukopaks were purchased from HemaCare. CD34+ hematopoietic stem cells (HSCs) were isolated using CliniMACS (Miltenyi) at UCLA. HSCs were thawed in warm R10 media and subsequently resuspended in dendritic cell (DC) differentiation media, which included Minimum Essential Medium alpha (MEMa; Gibco, Thermo Fisher Scientific, CAT# 12571063) with 20% defined FBS with 2X concentration of the following cytokines: 5 ng/ml stem cell factor (SCF; Peprotech, CAT# 300-07), 5 ng/ml FMS-like tyrosine kinase 3 ligand (FLT3-L, Peprotech, CAT# 300-19), 50 ng/ml thrombopoietin (TPO; Peprotech, CAT# 300-18), and 10 ng/ml granulocyte macrophage colony-stimulating factor (GM-CSF; Peprotech, CAT# 300-03). DMS79 cells were cultured in R10 media.

Generation of DCs

MS5-DLL1 cells were resuspended in MEMa (Thermo Fisher Scientific, CAT# 32561-037) supplemented with 20% defined FBS (GE Life Sciences, CAT# SH30070.01) and plated at 5,000 cells/well in 100 µL in a flat-bottom 96-well plate. The next day, CD34+ HSCs were thawed and resuspended in MEMa with 20% defined FBS with 2X concentration of the following cytokines: 5 ng/mL SCF (Peprotech, CAT# 300-07), 5 ng/mL FLT3-L (Peprotech, CAT# 300-19), 50 ng/mL TPO (Peprotech, CAT# 300-18), and 10 ng/mL GM-CSF (Peprotech, CAT# 300-03). HSCs were then plated on top of the MS5 cells at 5,000 cells/well in 100 µL media. Every 3-4 days, half of the media was replaced with fresh media and 2X cytokines. At 21 days after plating, cells were collected and CD45+ cells were isolated using CD45 microBeads (Miltenyi, CAT# 130-045-801). The DC phenotype was confirmed by flow cytometry using staining with CD66b-FITC (BioLegend, CAT# 305103), CD141-PerCP/Cy5.5 (BioLegend, CAT# 344111), and SIRPa-PE (BioLegend, CAT# 323806).

Autologous T-cell priming by DCs

When conventional type 1 dendritic cells (cDC1 cells) were used as antigen-presenting cells (APCs), autologous T cells were originally frozen down as the CD34- fraction of mobilized peripheral blood. On the day of DC harvesting, CD34- cells were thawed in warm R10 media and incubated overnight in AIM-V media supplemented with 5% heat-inactivated human AB serum, 1X GlutaMAX, and 55 µM β-mercaptoethanol with 5 ng/mL IL7 (Peprotech, CAT# 200-07). Isolated CD45+ DCs were resuspended in DC differentiation media with 10 µg/mL poly(I:C) (Sigma, CAT# P1530-25MG), 10 µg/ml R848 (Sigma, CAT# SML0196-10MG), and the peptide pool of interest, such that the concentration of each peptide was within 2-10 µg/mL. The DCs were then plated overnight to mature in a 96-well V-bottom plate at 200 µL/well. The next day, T cells were isolated using magnetic beads (Miltenyi, CAT# 130-096-535). Mature DCs and isolated T cells were combined at a 1:4 ratio in AIM-V media supplemented with 5% heat-inactivated human AB serum, 1X GlutaMAX, 55 µM β-mercaptoethanol, 5 µg/mL poly(I:C), 5 µg/mL R848, 10 ng/mL GM-CSF, 30 ng/mL IL21 (Peprotech, CAT# 200-21), and 5 ng/mL IL7. The cell suspension was then plated into 48-well plates at 500 µL/well. Three days later, cells were supplemented with fresh AIM-V media supplemented with 5% heat-inactivated human AB serum, 1X GlutaMAX, 55 µM β-mercaptoethanol, 10 ng/mL IL7, and 10 ng/mL IL-15 (Peprotech, CAT# 200-15). T-cell expansion in IL-7/IL-15 was carried on until analysis. Priming assay replicates were kept separate throughout the expansion phase to prevent dilution of antigen-specific responses.

T-cell priming by PBMCs

Frozen vials of primary human PBMCs from a single donor were purchased from AllCells (Alameda, CA). PBMCs were thawed and plated at 5 x 10^6 cells/mL in AIM-V media supplemented with 5% heat-inactivated human AB serum, 1X GlutaMAX, 55 µM β-mercaptoethanol, 50 U/mL IL-2, and 1 ng/mL in a 24 well plate. Cells were rested overnight. On

the following day, 1 mL of media containing 1-10 µg/mL peptide and 50 U/mL IL-2 was added. A half media change with 2X peptide and cytokine was performed every 2-3 days for up to 9 days.

Intracellular staining
For intracellular staining, T cells were washed 3 times with PBS and once with AIM-V media supplemented with 5% heat-inactivated human AB serum, 1X GlutaMAX, and 55 µM β-mercaptoethanol. Cells were plated into 96-well U-bottom plates at 200,000 cells/well in 100 µL of AIM-V media supplemented with 5% heat-inactivated human AB serum, 1X GlutaMAX, and 55 µM β-mercaptoethanol. At 12-17 hours later, 5-10 µg/mL peptide and 1 µg/mL CD28/49d antibodies (BD, CAT# 347690) were added in 100 µL. One hour later, 1X Brefeldin-A (BFA; BioLegend, CAT# 420601) was added. Cells were incubated for 8 more hours and were then stained for intracellular TNFα and IFNγ using the commercially available kit (BD, CAT# 554714). The following antibodies were used: CD3-APCCy7 (Thermo Fisher Scientific, CAT# 47-0036-42), CD8a-PE (Thermo Fisher Scientific, CAT# 12-0088-42), CD4-PECy7 (BioLegend, CAT# 300512), IFNγ-APC (BioLegend, CAT# 506510), and TNFα-FITC (BioLegend, CAT# 502906).

CD137 staining and sorting
T cells were prepared and stimulated as described above for intracellular staining. BFA (BioLegend, CAT# 420601) was excluded to allow for surface upregulation of CD137. Cells were stimulated for 24 hours and then stained for CD3-APCCy7 (Thermo Fisher Scientific, CAT# 47-0036-42), CD8a-PE (Thermo Fisher Scientific, CAT# 12-0088-42), CD4-PECy7 (BioLegend, CAT# 300512), and CD137-APC (BioLegend, CAT# 309810). Flow cytometry was performed, with cells gated on CD3+, CD8+/ CD4-, and CD137+ and sorted into an Eppendorf tube with 400 µl of 0.04% BSA for downstream TCR sequencing.

10X Genomics single-cell VDJ sequencing
The TCR VDJ libraries were constructed by the Technology Center for Genomics & Bioinformatics at UCLA per the standard 10X Genomics protocol. Libraries were then sequenced on MiSeq or NextSeq (Illumina).

Cloning of TCR constructs
TCR alpha and beta chain sequences from activated cells returned from 10x Genomics sequencing were ordered as gBlocks (IDT or Twist) and cloned as previously described (4).

TCR screening in Jurkat NFAT-GFP
TCRs were cloned into the pmaxCloning^{TM} Vector (Lonza, CAT# VDC-1040) and screened by co-culturing Jurkat-NFAT-GFP CD8 reporter cells and K562-A2. Miniprep plasmid DNA was purified (Qiagen, CAT# 27106) and eluted in nuclease-free water. DNA concentrations were routinely above 200 ng/µL. Jurkat cells were spun down at 596 x *g* for 5 min and resuspended in 20 µL of the Lonza SE cell line media (Lonza, CAT#: V4XC-1032) per transfection reaction. Next, 5×10^5 Jurkat cells and 2 µL of miniprep DNA were added per nucleofection well and electroporated using the 4D Nucleofector Jurkat E6.1 protocol (Lonza). Reactions were rested for 10 min, and then 80 µL of warm R10 media was added to each well and plated in 500 µL of R10 media overnight. The next day, cells were stimulated with K562-A2 cells loaded with DMSO (solvent control) or peptide at 10 µg/ml. Co-cultures were incubated overnight. The following day, 96-well plates were spun down at 1,026 x *g* for 2 min and stained with CD8-PE (Invitrogen, CAT# 12-0088-42) and murine TCR beta-APC (BioLegend, CAT# 109212) antibodies. The response was quantified using flow cytometry with the following gating scheme: light scatter, murine TCR+/CD8+, and GFP+.

Peptide pooling and deconvolution
Peptides were pooled in both a total pool and in a tiled sub-pool matrix, where peptides were represented in two unique sub-pools. TCRs reactive to two sub-pools were then re-stimulated with the corresponding single peptide. Peptide pool deconvolution and single peptide confirmation were performed using sub-pools of the total peptide pool and single peptide at 10 µg/mL.

PBMC activation and transduction

Frozen vials of primary human PBMCs from a single donor were purchased from AllCells. PBMCs were thawed and plated in 2 mL at 1.5 x 10^6 cells/mL with Dynabeads (Thermo Fisher Scientific, CAT# 11132D) at a 1:1 cell to bead ratio in AIM-V media supplemented with 5% heat-inactivated human AB serum, 1X GlutaMAX, 55 μM β-mercaptoethanol, 50 U/mL IL-2, and 1 ng/mL IL-15 for 48 hours. PBMCs were transduced by retroviruses encoding candidate TCRs. Viruses were thawed at 37°C. Media was removed from PBMCs and replaced with unconcentrated retroviral supernatant and 5 μg/mL polybrene (Sigma, CAT# H9268). PBMCs were centrifuged at 30°C for 90 min at 1,455 x *g*. After centrifugation, retroviral supernatant was replaced with media and incubated at 37°C overnight. Spinfection was repeated the following day. At 24 hours after the second spinfection, the cells and beads were washed with 1X PBS and re-plated with fresh AIM-V media supplemented with 5% heat-inactivated human AB serum, 1X GlutaMAX, 55 μM β-mercaptoethanol, 50 U/mL IL-2, and 1 ng/mL IL-15. At 48 hours after washing, the beads were removed by magnet (BD, CAT# 552811) and re-plated at 1.5 x 10^6 cells/mL. Cells were expanded for one week with intermittent half-media changes before use in functional assays. Experimental TCRs were prepared in the same batch of PBMCs as NGFR and F5 for use in functional assays. Reported experiments contain only cells from the same donor and spinfection.

PBMC co-cultures with K562-A2

PBMCs were combined with K562-A2 cells at a 2:1 effector to target ratio in a 96-well flat-bottom plate in 200 μL total of AIM-V media supplemented with 5% heat-inactivated human AB serum, 1X GlutaMAX, 55 μM β-mercaptoethanol, and 1 μg/m CD28/49d co-stimulatory antibodies (BD CAT# 347690, clones L293 and L25). DMSO solvent control was used as a negative control, and peptide was used at 10 μg/ml. In peptide dilution experiments, K562-A2 with cognate peptides were prepared at 10 μg/mL and diluted 10-fold seven times. Co-cultures were incubated at 37°C. At 48 hours post-plating, 20 μL of supernatant was removed and frozen at -80°C for IFNγ release measurement by ELISA. A positive control TCR (F5) and its cognate peptide (MART-1) were included in each experiment. The TCR clone F5 recognizes the HLA-A*02:01-presented epitope of MART-1 (ELAGIGILTV). All stimulations were done in triplicate. Real-time quantitative live-cell imaging was performed using the Incucyte system (Sartorius, Germany). Surface area of target cells was quantified based on GFP signals.

IFNγ release ELISA assay

After 48 hours of PBMC-K562 co-culture, the supernatant was collected for IFNγ analysis. IFNγ was quantified using sandwich cytokine ELISA OptEIA Reagent Set B (BD, CAT# 550534) and OptEIA human IFNγ ELISA kit (BD, CAT# 555142).

Supplementary References

1. T. Derrien, *et al.*, Fast Computation and Applications of Genome Mappability. *PLoS One* **7**, e30377 (2012).
2. T. U. Consortium, *et al.*, UniProt: the Universal Protein Knowledgebase in 2023. *Nucleic Acids Res.* **51**, D523–D531 (2023).
3. S. F. Altschul, W. Gish, W. Miller, E. W. Myers, D. J. Lipman, Basic local alignment search tool. *J. Mol. Biol.* **215**, 403–410 (1990).
4. Z. Mao, *et al.*, Physical and in silico immunopeptidomic profiling of a cancer antigen prostatic acid phosphatase reveals targets enabling TCR isolation. *Proc. Natl. Acad. Sci. U. S. A.* **119**, e2203410119 (2022).
